# DRGD-linked charged EKKE dimeric dodecapeptide: pH-based amyloid nanostructures and their application in lead and uranium binding†

**DOI:** 10.1039/d3ra08261j

**Published:** 2024-03-19

**Authors:** Aishwarya Natarajan, Late Ramakrishna Vadrevu, Krishnan Rangan

**Affiliations:** a Department of Chemistry, Birla Institute of Technology and Science Pilani, Hyderabad Campus Jawahar Nagar Hyderabad 500 078 Telangana India rkrishnan@hyderabad.bits-pilani.ac.in; b Department of Biological Sciences, Birla Institute of Technology and Science Pilani, Hyderabad Campus Jawahar Nagar Hyderabad 500 078 Telangana India

## Abstract

Peptides have been reported to undergo self-assembly into diverse nanostructures, influenced by several parameters, including their amino acid sequence, pH, charge, solvent, and temperature. Inspired by natural systems, researchers have developed biomimetic peptides capable of self-assembling into supramolecular functional structures. The present study explored a newly designed peptide sequence, EKKEDRGDEKKE, where E = glutamic acid, K = lysine, D = aspartic acid, G = glycine, and R = arginine, with a metal binding DRGD sequence incorporated between the exclusively charged EKKE peptide. We investigated the formation and the potential of the EKKEDRGDEKKE peptide in retaining the structure and morphology adopted by the individual EKKE peptide. According to a combination of experimental techniques such as thioflavin T fluorescence, field emission-scanning electron microscopy, atomic force microscopy, and circular dichroism, it was evident that the EKKEDRGDEKKE peptide displayed a pH-dependent propensity to adopt amyloid-like structures. Furthermore, the self-assembled entities formed under acidic, basic, and neutral conditions exhibited morphological variations, which resembled that observed for the exclusively charged EKKE peptide. Furthermore, the incorporation of the functional DRGD motif resulted in promising binding to two toxic metal ions, lead (Pb) and uranium (U), as evidenced by a range of spectroscopic techniques, including UV-visible spectroscopy, atomic absorption spectroscopy, fluorescence spectroscopy, and X-ray photoelectron spectroscopy. The use of the amyloid-forming EKKEDRGDEKKE scaffold can also be extended to potential biomedical applications.

## Abstract 2

Charged amino-acid containing dodecapeptide Ac-EKKEDRGDEKKE-CONH_2_ forms pH-based self assembly and amyloid nanostructures which binds heavy metals lead and uranium.

## Graphical abstract

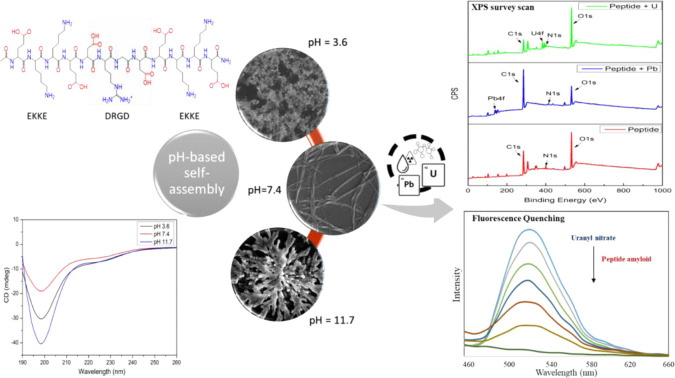

## 1 Introduction

Water contamination due to the accumulation of toxic metals is a growing concern for public health and the environment worldwide.^1,2^ The discharge of heavy metals from industrial wastewater, mining, agricultural, and anthropogenic activities is causing significant damage to the ecosystem and posing challenges to human health due to heavy metal poisoning.^3,4^ However, although remediation techniques such as chemical precipitation, ion exchange, membrane filtration, and biological remediation are currently employed, they have inherent drawbacks.^5,6^ In this context, the investigation of functional materials obtained from natural and sustainable sources for the purpose of metal-specific remediation has emerged as a significant field of research. Peptides/proteins from nature have become a source of inspiration for designing nanoscale materials based on the bottom-up approach.^7–9^ In nature, polypeptides represent examples of nanomaterials such as mineralized shells, bones, spider webs, silk, and curli (biofilms),^10–12^ which are highly ordered β-sheet amyloid fibers.^13,14^ Amyloid sequences are distinct β-sheet-forming structures, and the amyloid backbone consists of amino acid residues prone to aggregation.^15^ Amyloids form backbone hydrogen bonds, and the side chains of these amino acids can be used as templates for the design of functional amyloid materials^16–18^ due to their highly ordered hierarchy, excellent thermal stability,^19^ protease resistance,^20^ and ability to withstand harsh environmental conditions. Therefore, the combination of amino acid residues to retain the amyloid nature of the peptide, together with the addition of amino acid residues that impart functionality, is widely explored owing to its biocompatibility and diverse potential applications.^21–23^ Examples of the applications of amyloid-forming structures include carbon dioxide capture,^24^ nanodevices,^25^ metal nanowires,^26^ water purification,^27^ and removal of toxic heavy metals.^28–30^ Previously, amyloids derived from various protein sources ranging from β-lactoglobulin, bovine serum albumin (BSA), and whey protein to other plant-based proteins have been evaluated for their ability to remediate heavy-metal contaminated water.^27,31,32^

A wide range of metals, such as iron, copper, zinc, magnesium, nickel, molybdenum, and manganese, binds to proteins^33^ and is involved in many enzymatic reactions, reactions of biochemical pathways, electron-transport chain, and formation of extracorporeal structures such as silks and glues.^34,35^ Therefore, protein-metal binding information is of significant interest in recovering or remediating toxic metal ions, given that integrating residues or recognition motifs induces specificity towards metal binding.^36^ The use of biomimetic approaches for protein design and chelation of metals has led to the development of metal-accumulating systems that allow the control of size, morphology, composition, and orientation of materials for binding.^37–39^ In our recent study, we reported the preparation of a four-residue self-assembling peptide, EKKE, consisting of alternating positive and negative charges, which forms amyloid fibrils with lead binding ability.^40^ The RGD motif is known to form a β-turn when placed in the N-terminal, C-terminal, or in the middle of the protein sequence.^41^ In this attempt, we extended the EKKE peptide by adding the DRGD motif to enhance the metal binding capability of the self-assembling EKKE peptide sequence to the toxic metals lead (Pb) and uranium (U). Uranium and lead have similar coordination properties and form electrostatic interactions with hard-metal oxygen ligands similar to calcium.^42,43^ Thus, many peptides/proteins have been designed to capture these toxic metals. In the study by Lu Zhou *et al.*,^44^ they engineered a uranyl-binding protein to increase its metal binding sensitivity and specificity to uranyl ions with femtomolar affinity. They proposed that a well-folded protein scaffold decorated with special uranyl binding motifs (arginine, glutamic, and aspartic acid) would lead to selectivity for uranium over other metals. In other instances, several biomimetic approaches using model proteins and peptides have been developed to shed light on the uranium binding sites and affinities.^37,38,45,46^

**Scheme 1:**
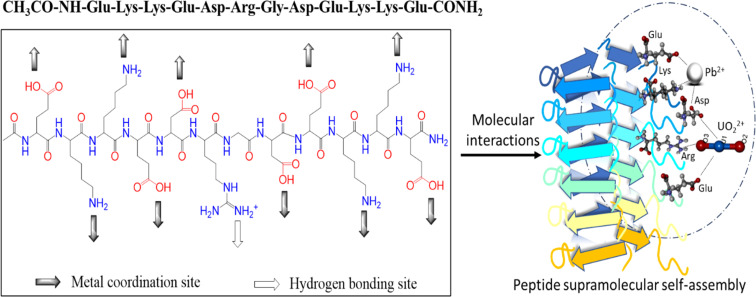
Novel-designed dodecapeptide Ac-EKKEDRGDEKKE-CONH_2_ and potential type of binding to lead and uranyl ions.

In this study, we investigated the potential impact of incorporating the particular amino acid residue DRGD into amyloid-forming EKKE sequences to enhance the functionality of EKKEDRGDEKKE dodecapeptide peptide amyloid-forming residues. The importance of this study is that it explores the effect of the incorporated additional amino acid residues consisting of arginine (R), which provide additional binding sites due to the presence of a positively charged H-bond donor group, where the charged N–H group of the arginine side chain acts as a hydrogen bond donor for metal ions, in particular the hazardous uranyl ion. This provides oxygen atoms as suitable hydrogen bond acceptors to attach to uranium, as shown in Scheme 1.

We focused on understanding how these charged amino acid residues may influence the fibrilization process and whether the resulting peptide will still exhibit the characteristic amyloid properties. The structural and morphological characteristics of the novel EKKEDRGDEKKE peptide, which consists of a DRGD sequence sandwiched by two EKKE sequences, were investigated in this study. Their amyloid-forming propensity were analyzed employing a thioflavin T (ThT) fluorescence assay, CD spectroscopy, SEM, and AFM. These techniques highlighted that the incorporation of the DRGD motif did not drastically alter the morphology and structure of the EKKEDRGDEKKE peptide. This observation emphasizes the significance of the EKKE peptide in the self-assembly process.^40^ The peptide amyloid material, EKKEDRGDEKKE, was further explored for its ability to bind to two toxic metals, lead (Pb) and uranium (U), to determine its metal binding propensity. We demonstrated that the affinity of lead acetate binding is more significant under alkaline conditions, as evidenced by the application of the UV-visible, X-ray photoelectron, and atomic absorption spectroscopy techniques. The self-assembled structures demonstrated notable sequestration of uranium ions under alkaline conditions, as investigated through UV-visible, fluorescence, and X-ray photoelectron spectroscopic methodologies.

## 2 Materials and methods

### 2.1. Materials

Ac-EKKEDRGDEKKE-CONH_2_ (CH_3_CO-NH-Glu-Lys-Lys-Glu-Asp-Arg-Gly-Asp-Glu-Lys-Lys-Glu-CONH_2_) peptide acetylated and amidated at the N- and C-termini, (theoretical average mass of 1531.63) with the empirical formula of C_62_H_106_N_20_O_25_ was purchased from Shanghai A Peptide with >95% purity. The peptide was purified on an HPLC system using 0.1% trifluoroacetic acid (TFA) in water and acetonitrile as the solvent system (Fig. S1†). The ESI-MS spectrum (Fig. S2†) confirmed the peptide structure with an *m*/*z* value of 1532.90 ([M + H]^+^, *z* = +1, predicted mass C_62_H_107_N_20_O_25_ 1532.63), 766.75 ([M + 2H]^2+^, *z* = +2) and 511.50 ([M + 3H]^3+^, *z* = +3). The studies were carried out with Milli Q water, and the chemicals thioflavin T (ThT), Arsenazo III, lead acetate, and uranyl nitrate were acquired from Sigma Aldrich, and HFIP (1,1,1,3,3,3-hexafluoro-2-propanol) from SRL Chem.

UV-visible spectroscopy, circular dichroism, and atomic absorption spectroscopic studies were performed on a JASCO V650 UV-vis, JASCO J1500 CD, and Shimadzu AA-7000 AAS spectrophotometer, respectively. The fluorescence spectroscopy studies were performed on a JASCO FP-6300 or SpectraMax iD3 (Molecular Devices). Scanning electron microscopy (SEM), atomic force microscopy (AFM), and X-ray photoelectron spectroscopy (XPS) data were obtained using an FEI Apreo LoVac Thermo Scientific FE-SEM, SPM Solver Nano AFM, and K-Alpha by Thermo Scientific XPS, respectively. A Nano-ZS Zetasizer by Malvern Instruments and Eutech Instruments were used for the zeta potential measurements and the pH potentiometric titrations, respectively.

### 2.2. Methods

#### 2.2.1 Preparation of peptide samples

Ac-EKKEDRGDEKKE-CONH_2_ (CH_3_CO-NH-Glu-Lys-Lys-Glu-Asp-Arg-Gly-Asp-Glu-Lys-Lys-Glu-CONH_2_) was obtained as a lyophilized powder, acetylated and amidated at the N and C termini, respectively. To remove any pre-formed aggregates, the lyophilized peptide was weighed and dissolved in 0.5 mL of 1,1,1,3,3,3-hexafluoro-2-propanol (HFIP) and incubated overnight. HFIP was later completely evaporated under vacuum using a Concentrator Plus (Eppendorf), leaving a thin peptide layer in the vial. Then, the peptide was dissolved in 1 mL of Milli Q water, and pH 3.6, pH 7.4, and pH 11.7 peptide solutions were prepared using HCl and NaOH, respectively. A Thermo Scientific NanoDrop One spectrophotometer was used to calculate the concentration of peptide, which was found to be 0.4 mg mL^−1^ by detecting its absorption at 205 nm and using the molar extinction coefficient of 31 mL mg^−1^ cm^−1^.^47^

#### 2.2.2 Thioflavin T fluorescence assay

**Fig. 1:**
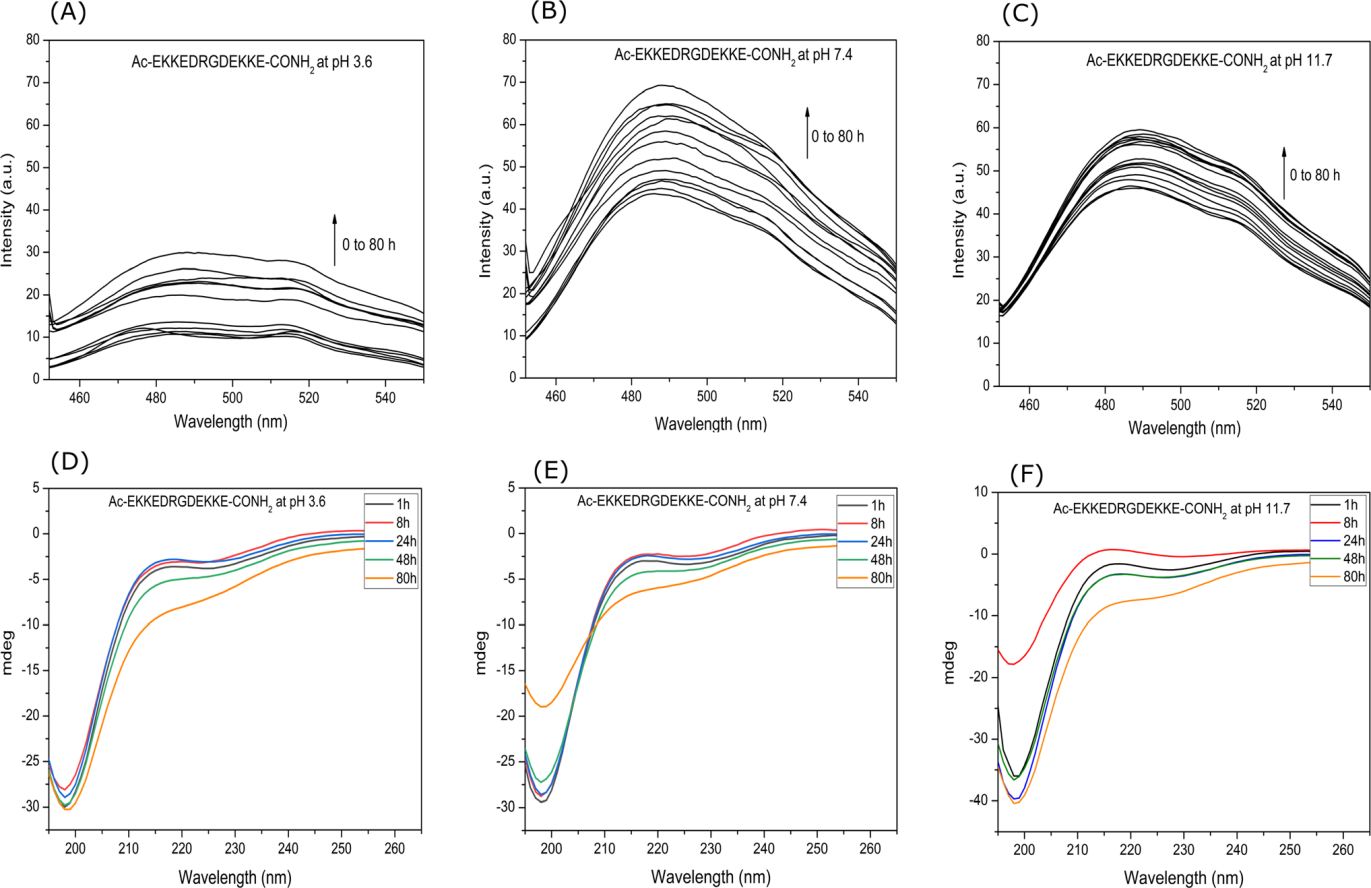
Enhanced ThT fluorescence assay and secondary structure estimation by circular dichroism study of Ac-EKKEDRGDEKKE-CONH_2_ peptide at three different pH values of 3.6, 7.4, and 11.7. Fluorescence enhancement of EKKEDRGDEKKE peptide treated with ThT at pH [A] 3.6, [B] 7.4, and [C] 11.7, over 0–60 h incubation period at the excitation wavelength of 450 nm. Secondary structure of the EKKEDRGDEKKE peptide characterized by circular dichroism spectroscopy at different pH conditions of [D] 3.6, [E] 7.4, and [F] 11.7.

A thioflavin T (ThT) stock solution was prepared by dissolving ThT (3 mg) in MilliQ water (10 mL) and filtering it with a 0.2 μm syringe filter. The concentration of the ThT stock solution was calculated by measuring the absorbance at 416 nm on a Jasco V650 UV-visible spectrophotometer and using the molar extinction co-efficient of 26 620 M^−1^ cm^−1^.^48^ The concentration of the prepared ThT stock solution was 9.4 × 10^−4^ M. Then, 1 mL of solution mixture containing 10 μL (9.4 × 10^−4^ M) of ThT solution and 100 μL (2.6 × 10^−4^ M) of peptide solution were prepared. Fluorescence spectra were recorded at different intervals for 26 μM EKKEDRGDEKKE peptide solution added to 9.4 μM ThT solution using a JASCO FP-6300 spectrofluorometer. The excitation wavelength was set at 440 nm and the slit width set to 5 nm for both the excitation and emission, and the emission intensities were measured in the range of 450 to 550 nm. All spectra are the buffer-corrected average of three accumulations (Fig. 1A–C).

#### 2.2.3 Circular dichroism spectroscopy

A Jasco J1500 CD spectrometer was used to measure the far-UV CD spectra for the peptide solutions (100 μM) at pH 3.6, 7.4, and 11.7. The CD spectra were recorded in the range of 250–195 nm using a cuvette with a 2 mm path length at 25 °C. An average of three measurements for each sample was performed, and the CD signals were measured at a scan rate of 100 nm min^−1^, with a data integration time of 4 s and a bandwidth of 2.5 nm.

#### 2.2.4 Dynamic light scattering

A Nano-ZS Zetasizer (Malvern Instruments Ltd, UK) was used to measure the particle size of the EKKEDRGDEKKE peptide. Briefly, 100 μM of the peptide was sampled at different pH levels (pH 3.6, pH 7.4, and pH 11.7) at room temperature in a fresh tube. A disposable cuvette with a path length of 1 cm was used. Each sample was subjected to three scans, each with 16 runs.

#### 2.2.5 Scanning electron microscopy

Field emission scanning electron microscopy (FE-SEM) was used to examine the formation and morphology of the self-assembled structures. 5 μL of the peptide samples at pH 3.6, 7.4, and 11.7 were deposited on a glass coverslip and dried for 30 min, and they were secured to a brass stub using carbon tape. A Leica Ultra Microtome EM UC7 Sputter coater was used to sputter-coat the samples with 6 nm gold at 20 mA for 1 min. The SEM images were captured at an accelerating voltage of 20 kV using an Apreo LoVac (FEI, Thermo Scientific). Lead acetate (10 ppm) and uranyl nitrate (10 ppm) solutions in water were independently incubated with 50 μM peptide amyloid (pH 11.7) solution overnight and the sample for EDAX measurement on a glass slide was prepared as above. An Aztec Standard EDS system with a resolution of 127 eV was used to perform the EDAX elemental mapping for the metal–peptide complex solutions.

#### 2.2.6 Atomic force microscopy

Similar to the process used for scanning electron microscopy, the samples for atomic force microscopy (AFM) were prepared by taking 5 μL of the peptide sample, casting it onto a glass coverslip, and letting it air dry for 30 min. An SPM Solver Nano with NT-MDT image processer was used to produce the AFM images. The atomic force microscope was operated in tapping mode at ambient temperature, with a scan window size adjusted to 20 cm^2^ to 90 cm^2^ and a scan rate of 0.8 Hz.

#### 2.2.7 Zeta potential measurements and pH potentiometric titration

The zeta potential studies for the peptide were carried out using a Nano-ZS Zetasizer. HCL (1 M) and NaOH (1 M) were added to obtain pH 2 to pH 12 peptide solutions, and the potential (mV) was measured in triplicate under various pH conditions. The pH of the peptide solutions during the potentiometric titrations was monitored using a glass microelectrode connected to an Eutech Instruments pH meter.

#### 2.2.8 X-ray fluorescence spectroscopy

Metal salts, lead acetate, uranyl nitrate, mercury chloride, cadmium chloride and vanadium sulphate were dissolved in water and stock solutions were made to 100 ppm. A 1 : 10 peptide and metal complex was obtained by adding 20 μL from 260 μM stock peptide solution to make 6 μM (10 ppm) peptide solution. To this solution, 980 μL of 100 ppm metal solution was added to a total volume of 1 mL. The metal–peptide complex was filtered using 3 kDa cut-off centrifugal filters, at 5000 rpm for 15 min. Further, the XRF profile of metal–peptide complex before and after filtration was obtained. Energy-dispersive X-ray fluorescence (ED-XRF) spectra of the metal–peptide complex were recorded on a Panalytical Epsilon-1 (M-788) instrument possessing an SDD5 detector. The parameters of the ED-XRF spectrum were maintained at a potential of 50 kV and an electric current of 100 mA.

#### 2.2.9 UV absorption spectroscopy for lead binding

UV absorption spectra were recorded on a Jasco V650 spectrophotometer in the wavelength range of 190–600 nm at room temperature, using a 1 cm path length cuvette and bandwidth of 1 nm. The spectra of lead(ii) acetate solution, EKKEDRGDEKKE self-assembled peptide solution, and peptide amyloid–lead solution were recorded in the same wavelength range. Further, 50 μM of self-assembled peptide amyloid solutions at pH 3.6, 7.4, and 11.7 were individually treated with 45 μM (15 ppm) of lead acetate solution. In addition, UV spectra were monitored at different lead concentrations (15, 30, 45, 60, 75, and 90 μM or 5, 10, 15, 20, 25, and 30 ppm) while maintaining the same peptide concentration (50 μM).

#### 2.2.10 Atomic absorption spectroscopy

The self-assembled peptide solutions (50 μM) were incubated at room temperature with 15 ppm lead acetate metal solution for 12 h at three pH values of pH 3.6, pH 7.4, and pH 11.7. The solutions were filtered and their filtrate analyzed for metal ion content using an AA-7000 atomic absorption spectrophotometer (Shimadzu). A metal solution of lead acetate with known concentrations was used to obtain a standard curve. Further, unknown concentrations were obtained for the incubated peptide–metal solutions.

#### 2.2.11 X-ray photoelectron spectroscopy (XPS)

The XPS studies for peptide and metal bound forms were performed on a Thermo Scientific K-Alpha instrument using an Al Kα X-ray, micro-focused monochromator source under a 1.5 × 10^−7^ mbar vacuum. The carbon corrections were performed using the Avantage software. 300 μL solution containing 50 μM of peptide and 45 μM lead acetate at pH 3.6, 7.4, and 11.7 were incubated overnight. A 300 μL solution containing 60 μM of peptide at pH 11.7 was incubated with 25 μM uranyl nitrate overnight for the uranium binding studies. 5 μL from this peptide–metal complex solution was added onto 5 mm × 5 mm glass slides. These slides were dried to obtain thin films and transferred to the XPS sample holder. The XPS survey scan spectra for each sample were recorded in the binding energy range of 0–1300 eV with an average of five scans. The narrow-band high-resolution spectra of Pb 4f, U 4f, N 1s, O 1s, and C 1s were acquired at an average of ten scans for quantitative binding energy measurements. The Fityk software was used for spectral deconvolutions.^49^

#### 2.2.12 Arsenazo-III assay for uranium binding

A 0.1% (1.29 mM) Arsenazo III (3,6-bis((*E*)-(2-arsonophenyl)diazenyl)-4,5-dihydroxynaphthalene-2,7-disulphonic acid) solution was prepared in water. The stock uranyl solution was made by dissolving 1 mg uranyl nitrate in 1 mL of water to obtain a 1000 ppm (2.53 mM) solution, and then diluted to obtain a stock solution of 100 ppm (0.253 mM). 20 μL of 1.29 mM Arsenazo III reagent was added to 5 ppm uranyl nitrate solution, and the final volume was made to 2 mL and was left to stand for 5 min. The absorbance was monitored in the visible range of 350 nm to 900 nm. The peptide amyloid–uranium binding studies were performed by incubating the uranyl Arsenazo complex with the EKKEDRGDEKKE amyloid peptide solution. 5 ppm uranyl nitrate (12.9 μM) + 12.9 μM of Arsenazo III dye (1 : 1) was added to 20, 40, 60, and 80 μM peptide and incubated overnight. The amyloid-uranium solution was filtered through a 3 kDa cut-off filter and centrifuged at 5000 rpm for 20 min. The absorption spectra of the filtrates were recorded to monitor the reduction of the uranium–Arsenazo complex concentration due to the binding with the amyloids.

#### 2.2.13 Fluorescence quenching study for uranium binding

Fluorescence quenching of uranyl nitrate (*λ*_ex_ = 266 nm, *λ*_em_ = 522 nm) by the peptide amyloids was performed on a microplate reader (SpectraMax iD3, Molecular Devices, SpectraMax software). Different concentrations of self-assembled (EKKEDRGDEKKE) peptide amyloids (5–70 μM) were added to 10 ppm (25 μM) of uranyl nitrate solution in microplate wells, and fluorescence spectra were measured the in the range of 450 nm–650 nm at the excitation wavelength of 266 nm.^30^ All measurements were performed in triplicate.

The fluorescence quenching process was analysed for both the static and dynamic quenching mechanism given that we expected the formation of a metal–peptide complex with the initial addition of the peptide.^50^ The Stern–Volmer quenching constant (*K*_SV_) was calculated from the linear region of the [*F*_0_/*F*] *vs.* [*Q*] plot at a lower concentration peptide following the Stern–Volmer equation,^50^ as follows:
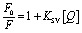
where *F*_0_ and *F* are the fluorescence intensity of uranyl nitrate solution in the absence and presence of peptide, respectively, and [*Q*] is the concentration of the peptide. The binding constant, *K*_A_, of uranium with peptide was obtained from the [(*F*_0_ − *F*_m_)/(*F*_0_ − *F*)] *vs.* 1/[*Q*] linear plot, where *F*_m_ represents the fluorescence intensity in the presence of excess peptide concentration, following a 1 : 1 binding profile and the Benesi–Hildebrand equation.^51^
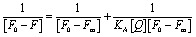


## 3 Results and discussion

### 3.1. Morphological and structural features of self-assembled EKKEDRGDEKKE peptide

**Fig. 2:**
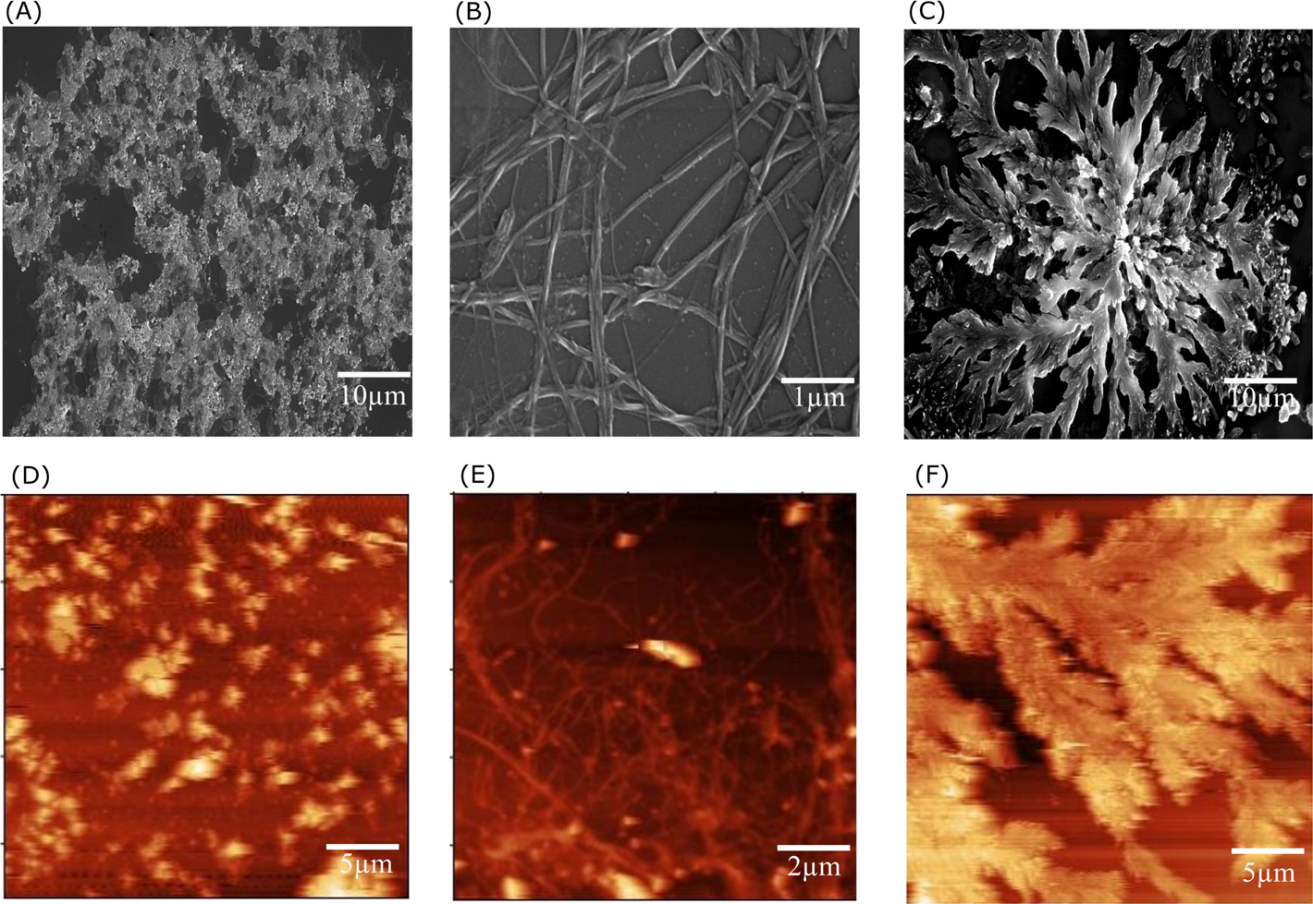
SEM and AFM images for the morphological study of Ac-EKKEDRGDEKKE-CONH_2_ self-assembled peptide using the FE-SEM and AFM techniques, respectively. FE-SEM images of EKKEDRGDEKKE at pH [A] 3.6, [B] 7.4, and [C] 11.7, and AFM images of the peptide at pH [D] 3.6, [E] 7.4, and [F] 11.7, where the images were recorded 7 days after sample preparation.

**Fig. 3:**
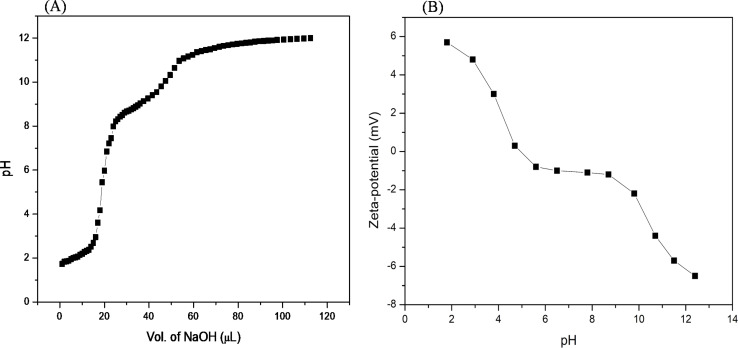
(A) pH titration of the acidified Ac-EKKEDRGDEKKE-CONH_2_ peptide (from pH 2.0) with 1 M NaOH. (B) Zeta-potential net charge (mV) *versus* pH of the Ac-EKKEDRGDEKKE-CONH_2_ peptide.

The propensity of the EKKEDRGDEKKE peptide (CH_3_CO-NH-Glu-Lys-Lys-Glu- Asp-Arg-Gly-Asp-Glu-Lys-Lys-Glu-CONH_2_) to form self-assembled amyloid structures was followed by observing the change in the fluorescence of thioflavin T (ThT) as a function of time. The time-based fluorescence enhancement spectra of aqueous solution mixtures containing 26 μM of EKKEDRGDEKKE peptide at different conditions of pH 3.6, 7.4 and 11.7, and 9.4 μM of thioflavin T dye were monitored. The fluorescence emission maximum of ThT shifted from 450 nm to 490 nm and was accompanied by an enhanced intensity, indicating the interaction of ThT with ordered beta-sheet peptide amyloid structures^52^ (Fig. 1A–C, respectively). The inherent property of the EKKE peptide to form amyloid-like structures was retained in the longer EKKEDRGDEKKE peptide.^40^ SEM and AFM images were recorded to study the amyloid-forming characteristic of this peptide, where 5 μL of 260 μM stock solution was drop-casted on a glass coverslip and dried under varying pH conditions. The SEM and AFM images of the peptide prepared at the acidic pH of 3.6 showed only the formation of amorphous structures (Fig. 2A and D). However, it formed ordered structures at pH 7.4 (Fig. 2B and E) and pH 11.7 (Fig. 2C and F). At pH 7.4, the peptide self-assembled into long-regular fibers, while at pH 11.7, branched nanofiber assemblies were observed. The branched assemblies of nanofibers observed at pH 11.7 exhibited similarities to the hierarchical fractal assemblies observed in the self-assembly and aggregation of many peptides/proteins.^53,54^ The width of the amyloid fibers formed by the Ac-EKKEDRGDEKKE-CONH_2_ peptide at pH 7.4 is around 0.06 μm, which was calculated by measuring the average width of the long regular nanofibers observed in the SEM image. Similarly, the width of the fractal morphological pattern formed at pH 11.7 was found to be around 0.1 μm. The formation of diverse morphological structures under acidic, neutral, and basic conditions for the EKKEDRGDEKKE peptide reflects its charge-based self-assembly as a result of the protonation/deprotonation states acquired by the peptide at pH 3.6, 7.4, and 11.7. The overall charges of the EKKEDRGDEKKE peptide will be +5, −1, and −5 at pH 3.6, 7.4, and 11.7, respectively, due to the deprotonation of the side chains of the E, K, and D amino acid residues under higher pH conditions (Fig. 3). Thus, the role of pH and charge were explored in the self-assembly of the peptide nanostructures. For instance, varying the number and repeats of the charged side-chain residue dictated the self-assembly of a class of peptides, *i.e.*, KFE12, KIE12, and KVE12. The side chains exhibited varying gelation behavior, which was influenced by the salt concentration at neutral pH.^55^ In another case, pH-responsive coiled-coil fibrils were obtained from AFD19 peptides, which appeared to form in-register coiled coils or offset fibrils depending on the pH and molecular charge of +1 or −1 suitable for gelation.^56^ Several examples emphasized the role of charge and pH in the self-assembly of peptides.^57–60^ The introduction of positively and negatively charged residues in a complimentary assembly may lead to the generation of nanofibers.^61–63^ The differential self-assembly of the EKKEDRGDEKKE peptide at pH 3.6, 7.4, and 11.7 strengthened the interplay among the charge, sequence, and nature of the amino acid residues.

The dynamic light scattering (DLS) profiles of the EKKEDRGDEKKE peptide, dissolved in pH 3.6, 7.4 and 11.7 solutions were assessed across different time points (1 h and 3 days) of their self-assembly. The mean size (*Z* average), which was calculated as a result of the auto-correlation function, demonstrated the presence of multiple size distribution and showed a slightly increasing trend as time progressed, as shown in Table S1.† The size profiles observed for pH 3.6 and 3 days of post incubation were 429.1, 432.0 and 596.9 nm with a polydispersity index (PdI) of 0.334. In the case of the pH 7.4 sample, the size profile values were 412.5, 465.8 and 570.0 with a PdI of 0.515, while for the amyloid peptide at pH 11.7, the corresponding size profile values were 413.1, 459.8, 552.5 with a PdI of 0.496.

The secondary structure compositions of the EKKEDRGDEKKE peptide at three distinct pH settings were analysed for 100 μM peptide solution over 0 h–80 h time period using circular dichroism (CD) spectroscopy at 25 °C. Amyloids and amyloid-like structures are commonly analysed using circular dichroism spectroscopy, which allows for the detection of their β-sheet secondary structure. This approach widely monitors the structural alterations in self-assembled peptide amyloid materials.

The circular dichroism spectra of EKKEDRGDEKKE at pH 3.6, 7.4, and 11.7 exhibited comparable signature patterns, featuring a dip at around 230 nm, a peak at about 215 nm, and a strong minimum signal at approximately 198 nm, as depicted in Fig. 1D–F, respectively.

The observation of a minimum at ∼230 nm and a positive peak or maximum at ∼215 nm suggests the presence of anti-parallel β-sheets.^64^ These features are similar to the characteristic circular dichroism spectra of the self-assembling peptide amyloid material EKKE peptide across three pH values (3.6, 7.4, and 11.7).^40^ The minima at ∼230 nm are also attributed to the presence of type-B fibrils, as reported in the low mechanical stress-induced glucagon fibril formation, having a positive peak at 203 nm and a negative peak at 230 nm.^65,66^ The presence of an additional strong signal at 198 nm corresponds to disordered or random coiled regions.^67^

Although the CD signature exhibited the common features for the EKKEDRGDEKKE peptide at the three different pH conditions, there were slight differences in its content of secondary structures at different pH conditions and different time points of recording the CD spectra. The secondary structure prediction tool BestSel^68^ was utilized to estimate the secondary structure content of the self-assembled EKKEDRGDEKKE peptide under three different pH conditions across different time points. At pH 3.6, it was observed that the disordered region constituted the majority of the structure, while the β-sheet content decreased and the α-helix content increased from 1 h to 80 h. Specifically, the α-helix content increased from 10.5% at 1 h to 21.9% at 80 h, whereas the β-sheet content decreased from 50.8% at 1 h to 39.6% at 80 h (Fig. S3A and B†). The proportion of disordered regions remained relatively stable, accounting for 38.7% at 1 h and 38.5% at 80 h.

The α-helix content in the peptide at pH 7.4 was initially 8.7% at 1 h, then increased to 16.7% at 80 h, and the corresponding β-sheet content of 52% at 1 h decreased to 37.7% over the next 80 h. The disordered regions at 1 h and 80 h for pH 7.4 peptide amyloids were 39.3% and 45.6%, respectively (Fig. S3B†). At pH 11.7, the α-helix content at 1 h was 14.5%, but no α-helix content was observed at 80 h. However, the β-sheet content increased to 51.5% at 80 h (Fig. S3†). The β-turn contribution for the overall composition under pH 7.4 and 11.7 conditions for the 80 h samples was 0% and 11.2%, respectively. The SEM and AFM images show thread-like morphologies at pH 7.4 and fractal pattern morphologies at pH 11.7. The final β-sheet contents of the peptide at pH 3.6, 7.4, and 11.7 were 39.6%, 37.7% and 51.5%, respectively (Table S2†). The α-helix secondary structure contents at pH 3.6, 7.4, and 11.7 conditions were 21.9%, 16.7% and 0%, and the corresponding disordered regions were 38.5%, 45.6% and 37.2%, respectively. In addition, at pH 11.7, 11.2% of β-turn regions was observed.

The spectroscopy and microscopy techniques were employed to study the morphologies of the self-assembled peptide nano-structures under similar conditions at pH 3.6, pH 7.4 and pH 11.7 and room temperature. The correlation in terms of the spectroscopic and microscopic techniques was based on the characteristic information derived from each technique. The presence of amorphous aggregates observed for Ac-EKKEDRGDEKKE-CONH_2_ at pH 3.6 from SEM and AFM was also corroborated by thioflavin T dye fluorescence spectra and CD, where a comparatively lower fluorescence fold change and beta sheet content were observed, respectively. Similarly, for Ac-EKKEDRGDEKKE-CONH_2_ at pH 7.4 and 11.7, thread-like or fibrillar structures and branched assemblies (fractal patterns) were observed, respectively, which were also supported by the thioflavin T fluorescence spectra with higher fluorescence intensity, indicating greater β-sheet formation.

**Scheme 2:**
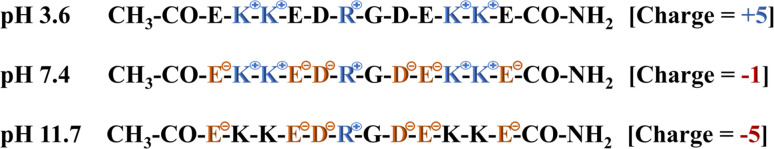
Charge of peptide at pH 3.6, 7.4 and 11.7.

To establish the interplay among charge, pH, and structure that the self-assembling EKKDRGDEKKE peptide attains under the three different pH conditions, pH titrations and zeta potential measurements were carried out (Fig. 3). The theoretical p*K*_a_ values for the side chains of charged amino acid residues were determined to be 3.65 and 4.25 for the acidic residues aspartic acid and glutamic acid, respectively. Similarly, the basic amino acid residues lysine and arginine exhibit theoretical p*K*_a_ values of 10.53 and 12.48, respectively. At pH 3.6, glutamic acid, lysine, and arginine are anticipated to be fully protonated due to their side chain p*K*_a_ values. However, aspartic acid, whose p*K*_a_ is close to the pH level studied, may not be entirely protonated. Consequently, the EKKEDRGDEKKE peptide carries a net charge ranging from approximately +4.5 to +5 relative to the NH_3_^+^ of the positively charged amino acids lysine (K) and arginine (R). Two sets of EKKE residues neutralize the net charge at pH 7.4. The overall charge is −1 due to the presence of one arginine residue with a side chain p*K*_a_ value of 12.48 (Scheme 2). At pH 11.7, the deprotonation of the acidic amino acid residues results in a net charge of −5 to −6, which is attributed to the side chain carboxylate groups on the acidic amino acids, specifically four residues of glutamic acid (E) and two residues of aspartic acid (D) (Fig. 3B). A potentiometric titration experiment was performed to determine the net charge of the self-assembled EKKEDRGDEKKE peptide. The pH range observed in the study encompassed values ranging from 1.7 to 12.0. The isoelectric point (pI) of the EKKEDRGDEKKE peptide was determined to be around 5.03. Additionally, the p*K*_1_, p*K*_2_, and p*K*_3_ values were discovered to be approximately 2.1, 8.4, and 10.9, respectively, as depicted in Fig. 3A.

The metal binding and sequestration capability of the EKKEDRGDEKKE peptide amyloids were studied for a mixture of metal ion solutions containing 10 equivalents of cadmium (Cd^2+^), mercury (Hg^2+^), lead (Pb^2+^), uranyl (U(O)_2_^2+^) and vanadyl (V(O)^2+^) ions. The metal ion binding preference of the EKKEDRGDEKKE peptide amyloids at pH 11.7 followed the order of uranyl > lead > cadmium > vanadyl > mercury, *i.e.*, greater preference for uranyl and lead ions (Fig. S4:† XRF of mixed metal ions before and after treatment with peptide amyloids, Table S3:† relative concentrations of cadmium (Cd^2+^), mercury (Hg^2+^), lead (Pb^2+^), uranyl (U(O)_2_^2+^) and vanadyl (V(O)^2+^) ions in solution before and after incubation with the EKKEDRGDEKKE peptide amyloids at pH 11.7).

**Fig. 4:**
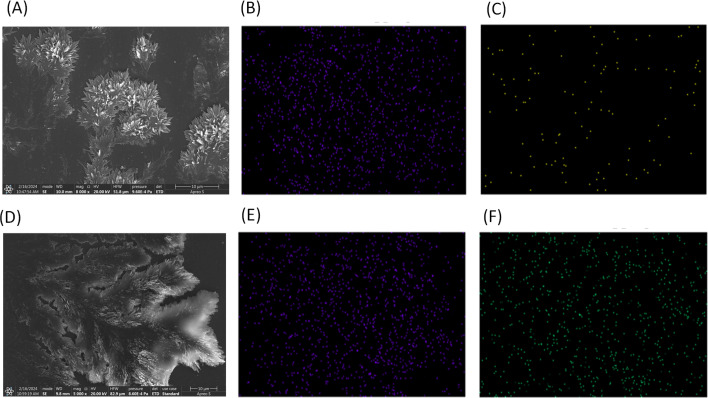
SEM images and SEM-EDAX elemental mapping of metal-EKKEDRGDEKKE amyloid peptide complexes prepared at pH 11.7. (A–C) SEM image and carbon and lead elemental mapping of lead–peptide complex, respectively. (D–F) SEM image and carbon and uranium elemental mapping of uranyl–peptide complex, respectively.

The SEM images of the Ac-EKKEDRGDEKKE-CONH_2_ peptide amyloids at pH 11.7 incubated with lead acetate and uranyl nitrate for studying their metal ion binding capability and detecting morphological changes (if any) were recorded, where the images of the metal-bound peptide amyloid complexes were compared to the peptide amyloid under identical conditions. Lead acetate (10 ppm) and uranyl nitrate (10 ppm) solutions in water were independently incubated with 50 μM peptide amyloid (pH 11.7) solution overnight. Then, 5 μL of the metal–peptide amyloid complex solutions were drop-casted on a glass coverslip and dried for 30 min for the SEM and SEM-EDAX study. The SEM images of the lead–peptide complex amyloid and uranyl–peptide complex amyloid are displayed in Fig. 4A and D, respectively. Although the morphologies of the peptide amyloid and the metal-bound peptide amyloid are relatively similar in nature, the uranium–peptide amyloid complex system also exhibited a fractal type of pattern, slightly opened in the third dimension along the viewing axis, and interestingly the SEM image of the lead-peptide amyloid complex showed a more leaf-like fractal morphology. Further, SEM-EDAX elemental mapping was performed to understand the distribution of metal ions in the amyloidal structures. The carbon EDAX and metal ion EDAX elemental mapping for the lead-peptide amyloid complex and uranyl-peptide amyloid complex are depicted in Fig. 4B, C, E and F, respectively. The elemental mapping indicated a close to evenly distributed metal ion complex centres throughout the amyloid soft-materials.

### 3.2. Interaction of lead with EKKEDRGDEKKE amyloids

#### 3.2.1 XPS spectroscopy studies

**Fig. 5:**
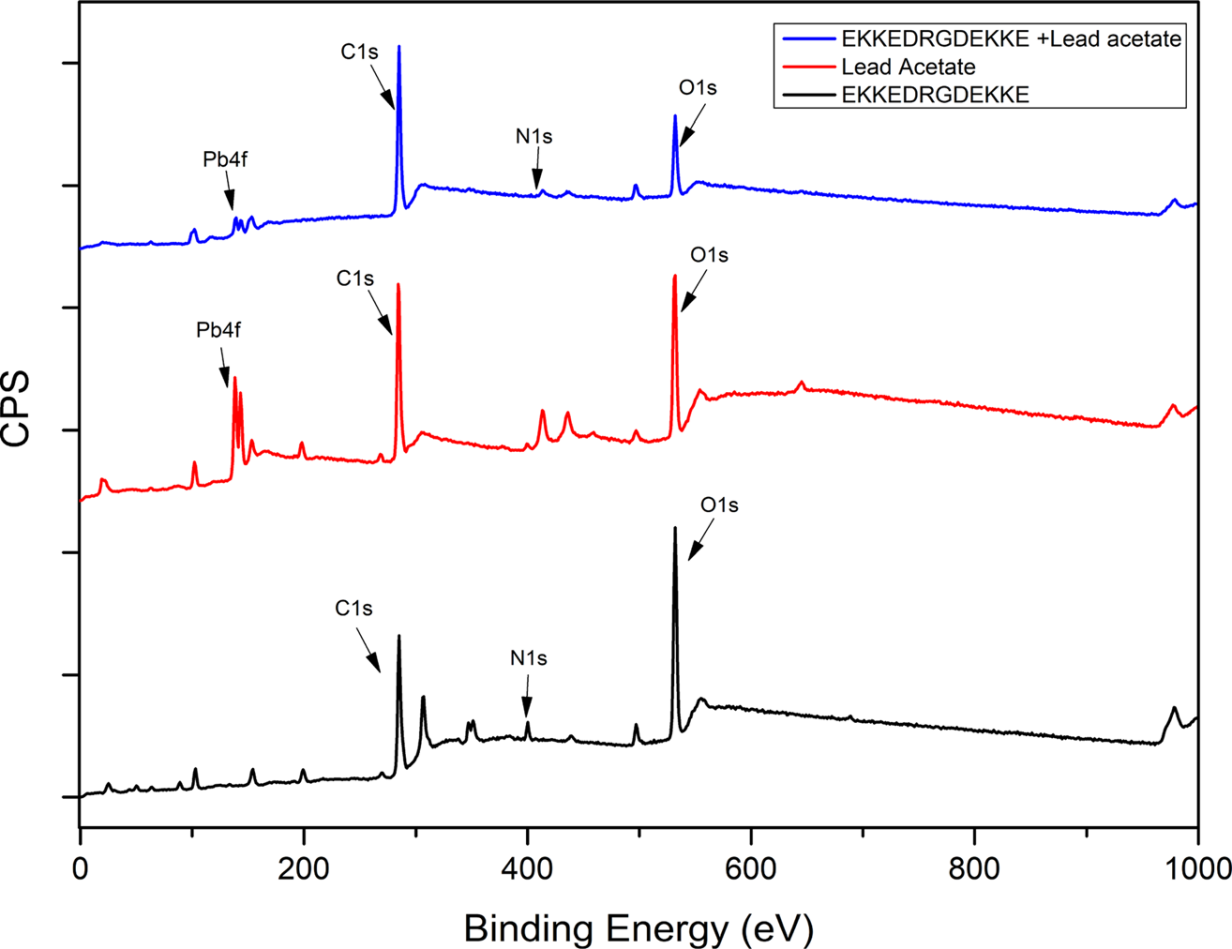
XPS survey spectra for EKKEDRGDEKKE peptide alone (pH 11.7), lead acetate, and EKKEDRGDEKKE peptide–lead complex (pH 11.7).

**Fig. 6:**
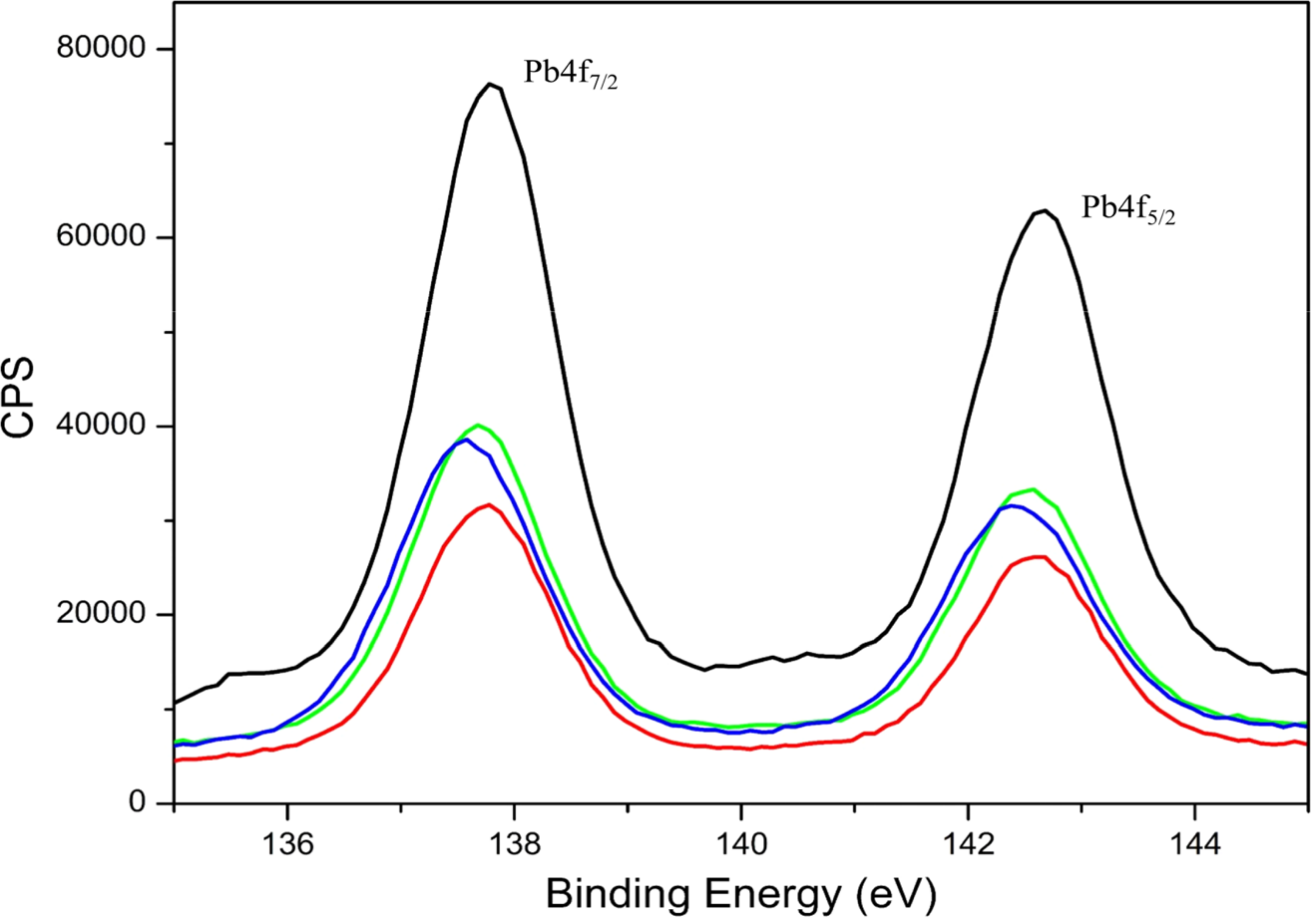
Narrow scan Pb XPS 4f_5/2_ and 4f_7/2_ spectra of lead acetate alone (black) and lead metal ion-EKKEDRGDEKKE peptide amyloid complex under different pH conditions of pH 3.6 (red), 7.4 (green), and 11.7 (blue). The XPS bands centered at around 142 eV and 138 eV correspond to 4f_5/2_ and 4f_7/2_, respectively.

The lead (Pb) metal ion binding to the EKKEDRGDEKKE peptide was monitored through XPS, UV-visible, and atomic absorbance spectroscopy instrumental methods for the peptide treated with lead under different conditions. The XPS survey scan demonstrated the presence of carbon (C), nitrogen (N), and oxygen (O) atoms for the EKKEDRGDEKKE self-assembled peptide amyloid material. The XPS survey scan of only lead acetate illustrated the presence of Pb, C, and O. Alternatively, the peptide–lead complexes were distinguished by the existence of binding energy (eV) associated with lead (Pb) features in addition to carbon (C), nitrogen (N), and oxygen (O), as shown in Fig. 5 and S5.† The narrow scan XPS of the Pb 4f regions was measured to study the interaction of lead with the peptide, as shown in Fig. 6. The peak at around 142 eV can be assigned to Pb 4f_5/2_, while the peak at around 138 eV is due to Pb 4f_7/2_.^69^ In the case of the free lead acetate, the Pb 4f_7/2_ and Pb 4f_5/2_ binding energy peaks were observed at 137.76 eV and 142.63 eV, respectively. The corresponding Pb 4f_7/2_ and Pb 4f_5/2_ peaks shifted to lower binding energies for the lead acetate-treated peptide, indicating the interactions of lead with the EKKEDRGDEKKE amyloid structures at the three different pH values, as shown in Table 1. The Pb 4f_7/2_ binding energy for the peptide amyloid-lead system at pH 3.6, 7.4, and 11.7 correspondingly appeared at 137.73 eV, 137.65 eV, and 137.52 eV, respectively. The lowest binding energy at the basic pH suggests the strongest EKKEDRGDEKKE–lead interaction at pH 11.7.

**Table 1:** The XPS Pb 4f binding energy (eV) values for lead acetate and EKKEDRGDEKKE amyloid–lead complex at pH 3.6, 7.4, and 11.7

Sample	Binding energy (eV)	*Δ*_metal_ (eV)
Lead acetate	137.76 (Pb 4f_7/2_), 142.63 (Pb 4f_5/2_)	4.87
EKKEDRGDEKKE (pH 3.6) + lead acetate	137.73 (Pb 4f_7/2_), 142.60 (Pb 4f_5/2_)	4.87
EKKEDRGDEKKE (pH 7.4) + lead acetate	137.65 (Pb 4f_7/2_), 142.52 (Pb 4f_5/2_)	4.87
EKKEDRGDEKKE (pH 11.7) + lead acetate	137.52 (Pb 4f_7/2_), 142.39 (Pb 4f_5/2_)	4.87

The narrow scan C 1s, N 1s, and O 1s XPS spectra were deconvoluted (Fig. S6–S8†) to further reveal the environment of the C, N, and O atoms under different pH and lead-treated conditions by exploring the binding energy differences between the self-assembled amyloid peptide in comparison to the lead–peptide amyloid complex, respectively. According to the C 1s XPS spectrum, we could interpret the binding of lead through the carboxylate groups of the peptide (Fig. S6†). Under the acidic condition of pH 3.6, the C 1s XPS peaks for the EKKEDRGDEKKE peptide were observed at 284.8 eV, 286.7 eV, and 288.9 eV, where the peak at 284.8 eV is due to hydrocarbon C atoms, 286.7 eV is due to the amine (**C**–N) and alpha carbon (N–CO–**C**–N–CO), and 288.9 eV is due to the amide ((**C**=O)–N) C atoms and carboxylic acid (O=**C**–OH) C atoms. Under the basic conditions of pH 7.4 and pH 11.7, three C 1s XPS peaks are expected for the EKEKDRGDEKKE peptide, which are (i) 284.8 eV due to the hydrocarbon C atoms, (ii) 286.8 eV due to the amine (**C**–N) and alpha carbon (N–CO–**C**–N–CO) and carboxylate (**C**OO–) C atoms, and (iii) 288.7 eV due to the amide ((**C**=O)–N) C atoms. (Fig. S6A, C, and E†), respectively. The carboxylate (O=**C**–O^−^) C(1 s) may have a lower binding energy than the carboxylic acid (O=**C**–OH) group C 1s atoms (Table S4†).

The EKKEDRGDEKKE peptide is expected to show carboxylic acid and carboxylate ion group-based O 1s XPS peaks due to the presence of aspartic acid and glutamic acid residues in addition to the peptide bond carbonyl groups (Fig. S7†). At pH 3.6, the peptide O 1s XPS peak appeared at 530.5 eV, 532.2 eV, and 534.1 eV in the deconvoluted spectrum, as shown in Fig. S7A.† These peaks are assigned to the carboxylic acid (**O**=C–OH) group carbonyl oxygen atoms, amide (**O**=C–NH) group carbonyl oxygen atoms, and carboxylic acid (H**O**–C=O) carbon-oxygen single bond oxygen atoms, respectively.^70^ In the case of the peptide at pH 7.4, the O 1s peaks were detected at 531.7 eV and 535.3 eV (Fig. S7C†), and the corresponding peaks appeared at 532.0 eV and 534.4 eV for the pH 11.7 peptide amyloid samples (Fig. S7E†), respectively. These peaks correspond to the amide (**O**=C–NH–) and carboxylate-Na^+^ functional groups, respectively. The O 1s XPS features for the peptide and peptide treated with lead acetate at pH 3.6 were almost similar, indicating less binding of lead at this acidic pH (Fig. S7B and Table S3†). The O 1s spectra of the EKKEDRGDEKKE–lead complex exhibited lead metal-bound carboxylate group XPS peaks at 535.4 eV and 535.0 eV for pH 7.4 and pH 11.7 (Fig. S7D and F†), respectively (Table S5†).

In the case of the lysine-containing peptides, the N 1s XPS binding energy peaks at around 399.3, 399.8, and 402.0 eV are ascribed to the contribution from the amine (C–**N**H_2_), amide (O=C–**N**H–C) and protonated amine (positively charged ammonium ion) (C–**N**H_3_^+^) groups, respectively.^70,71^ The narrow band N 1s XPS spectra of the EKKEDRGDEKKE peptide at acidic pH 3.6 showed binding energy peaks at 399.8 eV and 402.5 eV, which are assigned to the amide (O=C–**N**H–C) groups of the peptide bonds and protonated amine group (R–**N**H_3_^+^) groups of the side chain lysine and arginine groups, respectively (Fig. S8A†). At pH 7.4, the XPS peak at 399.7 eV, the most intense peak, is attributed to the amide N atoms. The peaks at 398.1 eV and 401.6 eV are assigned to the lysine side chain of the peptide, with the former representing the small faction of deprotonated –NH_2_ form and the latter indicating the relatively higher presence of protonated –NH_3_^+^ groups under slightly basic conditions. This assignment is based on the knowledge that the p*K*_a_ value of the lysine side chain amino group is 8.9 (Fig. S8C†). In the case of the EKKEDRGDEKKE peptide alone at pH 11.7, the N 1s peaks appeared at 397.8 eV and 399.7 eV, corresponding to amine (C–**N**H_2_) and amide (O=C–**N**H–C), respectively (Fig. S8E and Table S4†). In the case of the lead-bound peptide at pH 7.4, the amide nitrogen group and the protonated/lead-bound amine group appeared at 399.6 eV and 402.0 eV, respectively (Fig. S7D†). For the lead-bound peptide at pH 11.7, the N 1s XPS peaks observed at 397.6 eV, 399.6 eV, and 402.0 eV are assigned to the deprotonated amine groups, amide groups, and the lead metal-coordinated amine groups, respectively (Fig. S8F and Table S6†).

Overall, the binding energy shifts for the EKKEDRGDEKKE peptide treated with lead in the XPS spectra are more prominent in the case of pH 7.4 and 11.7 compared to pH 3.6. Similar observations were also seen in the case of the Pb 4f spectra, suggesting the change in the electronic environment of lead upon binding to the peptide (Fig. 6). The shift in the binding energy was higher at pH 7.4 and 11.7. The C 1s, N 1s, O 1s, and Pb 4f spectra complimentarily indicate the self-assembled peptide-lead interactions and that the interacting atoms with lead are oxygen and nitrogen. The carboxylate and amine groups present in the peptide act as chelating ligands that bind efficiently with the metal ion. Therefore, Pb^2+^ is more easily complexed with the carboxylate (–COO^−^) and the amine (–NH_2_) groups on the surface of the self-assembled peptide material. Based on the C 1s, N 1s, and O 1s XPS spectral study, the peptide binds to lead through the interaction of Pb^2+^ with the oxygen of the carboxylate groups of glutamic/aspartic acid and the nitrogen of the lysine side-chain amino group. At pH 7.4, the interaction is probably mainly from the carboxylate groups and lead. However, at the higher pH of 11.7, the deprotonated amino group of the lysine side chains is also available for binding with Pb^2+^.

#### 3.2.2 UV absorption spectroscopy

**Fig. 7:**
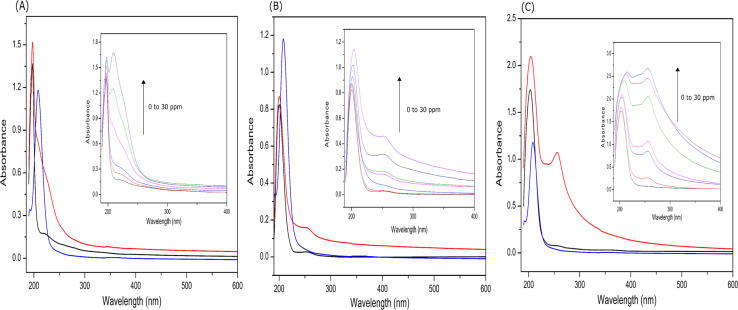
UV-visible absorption spectra of EKKEDRGDEKKE peptide with incremental addition of lead acetate at the three different pH of 3.6 (A), 7.4 (B), and 11.7 (C). The black line (50 μM EKKEDRGDEKKE peptide), blue line (15 ppm lead acetate), and red line (50 μM EKKEDRGDEKKE + 15 ppm lead acetate complex). The insets show the spectra of the EKKEDRGDEKKE peptide titrated with 0 to 30 ppm of lead acetate at pH 3.6, 7.4, and 11.7, respectively.

Fig. 7 shows the UV absorption spectra for the EKKEDRGDEKKE peptide and lead(ii) acetate binding to the peptides under pH 3.6, 7.4, and 11.7 conditions. Lead(ii) acetate is reported to exhibit a signal between 205 and 215 nm for Pb^2+^ in the UV region.^72^ The peptide in the absence of lead shows π–π* electronic transitions from the peptide backbone for self-assembled peptide amyloid solutions at around 200 nm.^73,74^ The interaction of the lead(ii) acetate with the self-assembled amyloid is apparent, where a new shoulder at ∼260 nm arises due to the ligand-to-metal charge transfer (LMCT) transition band.^39,75,76^ Further, it should be noted that the signal at 260 nm showed a concentration-dependent and pH-dependent intensity change (Fig. 7B and C, inset), which was more prominent at pH 7.4 and 11.7, respectively. The metal interaction was also evident given that the signal at 260 nm increased with an increase in the metal concentration from 0–30 ppm lead acetate solution. The peak at 260 nm appeared to be more pronounced for the EKKEDRGDEKKE peptide amyloid at pH 11.7, indicating better amyloid-Pb^2+^ binding, which is consistent with the observed shifts for lead upon binding. This observation is also consistent with the shifts in the XPS spectra observed for the EKKEDRGDEKKE–lead complex at pH 7.4 and 11.7 (Fig. 6). The Pb(ii)–peptide complex exhibited a lead–peptide interaction due to the appearance of a peak at ∼260 nm in the lead acetate and peptide solution, which possibly arises from the peptide coordination with the metal ion due to charge attractions (aspartic and glutamic acid). The presence of side-chain carboxylate and amine groups of the amino acids from the self-assembled peptides served as electron donors for participation in the metal ion chelation. Asp and Glu are known to be involved in lead metal ion binding, and being negatively charged at pH 7.4 and 11.7, the deprotonation of the carboxylic acid side chain groups seemingly also contributes to the binding of the positively charged lead metal ion.^39^

The information obtained from X-ray photoelectron spectroscopy and UV-absorption spectroscopy establishes the interaction of the self-assembled peptide amyloid material with the lead metal ion. The observed interaction between the self-assembled peptide material and the lead metal suggests that it is not solely a physical adsorption process. This inference is supported by the observed shifts in the binding energy values (electron volts) for the lead metal ion, as indicated in Table 1 and Fig. 6. Additionally, the changes observed in the C 1s, O 1s, and N 1s binding energy spectra and the presence of an LMCT peak in the UV-absorption spectra further indicate the formation of a complex between the peptide and metal ions in the solution.

#### 3.2.3 Atomic absorption analysis

The reduction in the overall concentration of lead metal is also evident in the results obtained from the atomic absorption spectroscopic analysis of the self-assembled peptide amyloid materials subjected to incubation with lead acetate solution. Evaluating lead metal binding using AAS offers a valuable approach to examining the affinity of metal ions towards the self-assembled EKKEDRGDEKKE amyloid fibrils. The lead acetate solution incubated with the self-assembled EKKEDRGDEKKE peptide amyloid material under three different pH conditions was filtered through a 3 kDa cut-off membrane filter. The filtrates were analyzed for the unbound lead ion contents. The graphical representation in Fig. 7 illustrates the quantity of lead acetate that remained in the solution after filtration. The lead binding capacity of the EKKEDRGDEKKE dodecapeptide was compared with that of the EKKE tetrapeptide. The EKKEDRGDEKKE peptide exhibited varying levels of lead binding under different pH settings, resulting in a varying retention of lead acetate in the solution. The unbound lead ion concentration in the pH 3.6 lead–peptide mixture filtrate was determined to be 66%.

**Fig. 8:**
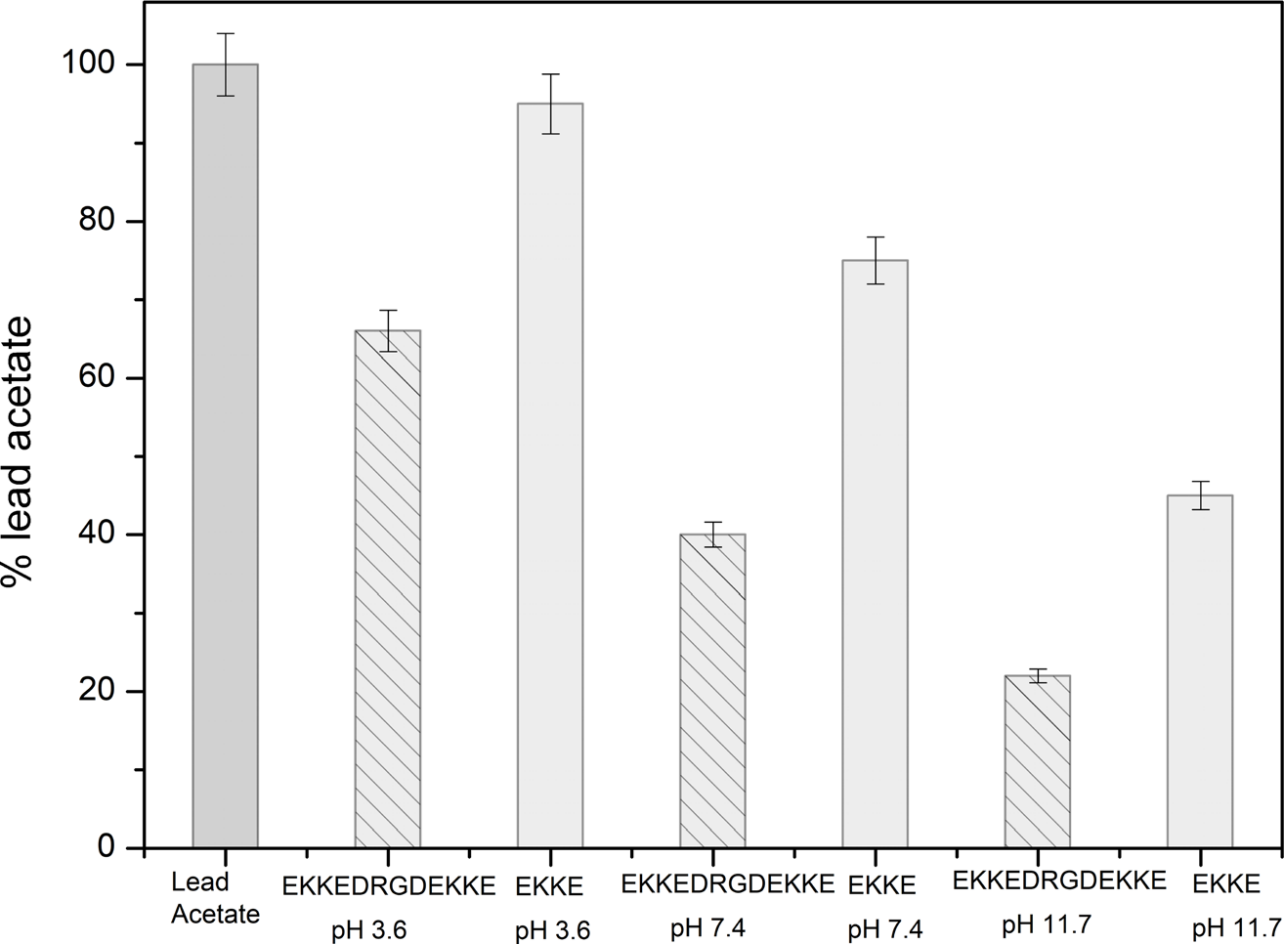
Concentrations of lead acetate based on atomic absorption spectroscopy before and after filtration through the self-assembled peptide amyloid materials EKKEDRGDEKKE and EKKE at pH 3.6, 7.4 and pH 11.7, where percentage lead represents the amount of lead present in the filtrate. A higher percentage denotes less retention on the amyloid material.

Consequently, the absorption of lead metal ions at pH 3.6 was estimated to be approximately 34%. At pH 7.4, the concentration of lead metal ions in the filtrate was around 40%, resulting in the corresponding absorption by the peptide amyloid of approximately 60%. This uptake is roughly twice that of the EKKEDRGDEKKE material at pH 3.6. The lead metal ion uptake reached the maximum value of approximately 78% at pH 11.7, with only around 22% of the lead metal ions detected. This observation suggests the stronger binding affinity of the peptide amyloids formed at pH 11.7 compared to the lower pH values. The EKKEDRGDEKKE peptide exhibited varying efficiency in binding to lead metal ions, resulting in a progressive reduction in the lead metal content. Specifically, the decreasing lead metal concentration followed the order of pH 3.6 < pH 7.4 < pH 11.7 (Fig. 8). The lead binding capacity of the EKKEDRGDEKKE dodecapeptide under all the pH conditions was found to be better than that of the EKKE peptide.

The results obtained from the atomic absorption spectroscopy analysis support the observations in the XPS and UV spectroscopic investigations, indicating the stronger affinity for lead binding at the alkaline pH of 11.7. This enhanced binding is likely attributed to the availability of deprotonated carboxylic acid and amine group residues at this pH, creating a favorable environment for the interaction with the positively charged metal ion.

### 3.3. Uranium binding to self-assembled EKKEDRGDEKKE peptide

Based on the above amyloid forming nature along with the lead metal ion binding tendency of the EKKEDRGDEKKE peptide at a basic pH medium, the following uranium binding studies on this peptide amyloid materials were carried out at pH 11.7 condition monitored through UV-visible, fluorescence, and X-ray photoelectron spectroscopy.

#### 3.3.1 Arsenazo dye absorption spectroscopy

**Fig. 9:**
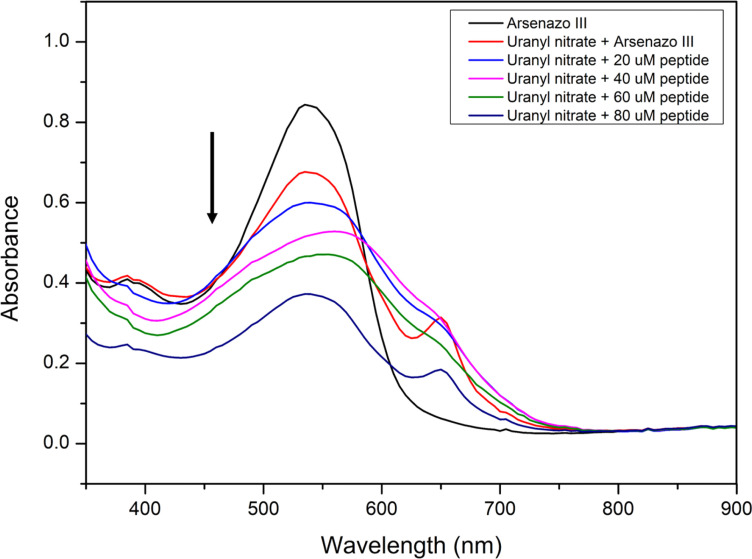
UV-visible absorption spectra of Arsenazo III dye in water (black), 12.5 μM (1 : 1 complex) of uranyl nitrate–Arsenazo III dye complex (red), uranyl nitrate (5 ppm) incubated with EKKEDRGDEKKE peptide amyloid complex (pH 11.7) at different concentrations, 20 μM (light blue), 40 μM (magenta), 60 μM (green) and 80 μM (dark blue).

UV-visible absorption spectroscopy was performed to study the binding of uranyl nitrate to the self-assembled EKKEDRGDEKKE peptide amyloid material at pH 11.7. A bis-azo chromogenic reagent, Arsenazo III, owing to its complexation tendency with rare-earth metal ions producing different color complexes, was systematically used to determine metal ions.^77,78^ The structure of Arsenazo III dye facilitates the formation of a 1 : 1 complex between Arsenazo III and uranium.^79,80^ The absorption peak at 553 nm corresponds to the Arsenazo dye absorption, and the complexation between uranyl(VI) nitrate and Arsenazo III dye was indicated by the additional absorption band at 653 nm, as seen in Fig. 9. The loss or imbalance in the stoichiometry between the metal and dye complex can lead to a shift or disappearance in the signal at 653 nm.^78^ Different amounts of EKKEDRGDEKKE peptide amyloid solutions were incubated with Arsenazo–uranium complex solution for 12 h. The filtrate of these mixtures was subjected to UV-visible spectroscopic studies and it was found that the absorbance intensity at 653 nm, which corresponds to the concentrations of Arsenazo–uranium complex, decreased with an increase in the concentration of the peptide–amyloid solution, indicating uranium binding and retention on the peptide amyloid material surfaces.

#### 3.3.2 Uranium fluorescence emission spectroscopy

**Fig. 10:**
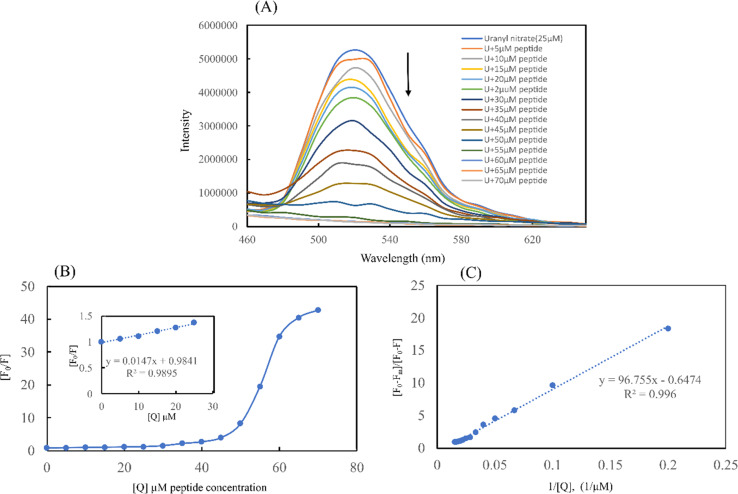
Fluorescence emission quenching of uranyl nitrate by EKKEDRGDEKKE peptide. [A] Fluorescence emission quenching of uranyl nitrate solution (10 ppm) titrated with an increasing concentration of EKKEDRGDEKKE self-assembled peptide (5, 10, 15, 20, 25, 30, 35, 40, 45, 50, 55, 60, 65, 70 μM). [B] Plot of differences in fluorescence intensity of uranyl nitrate at 520 nm *vs.* concentration of EKKEDRGDEKKE peptide. (Inset) Stern–Volmer plot of fluorescence quenching at lower concentration of EKKEDRGDEKKE self-assembled peptide amyloid to determine the fluorescence quenching constant *K*_SV_. [C] Benesi–Hildebrand plot of [(*F*_0_ − *F*_m_)/(*F*_0_ − *F*)] (*F*_0_ is the fluorescence intensity without the peptide and *F*_min_ is the minimum fluorescence intensity upon quenching) *vs.* [1/*Q*] (*Q* = peptide concentration in μM) showcasing 1 : 1 metal and peptide binding.

Fluorescence spectroscopy was conducted to provide additional evidence of the uranium binding. Fluorescence emission spectra were recorded for uranyl nitrate solution titrated with increasing concentrations of self-assembled EKKEDRGDEKKE amyloid peptide. The steady decrease in fluorescence emission intensity for the uranyl peak at 520 nm^30^ was observed as the peptide concentration increased from 5 to 70 μM (Fig. 10A). No significant emission *λ*_max_ shift was observed for the self-assembled EKKEDRGDEKKE peptide–uranyl complex solutions. The observed decline in the fluorescence signal of uranyl nitrate with an increasing concentration of the peptide material is consistent with the UV-visible absorption spectra acquired using the Arsenazo-III assay for uranium binding. Upon the addition of the peptide-amyloid material to uranyl nitrate solution, it was observed that at concentrations of up to 50 μM, a discernible amount of uranium was present, in terms of the intensity observed at 520 nm. However, at a 70 μM concentration of the peptide-amyloid solution, the signal declined notably, leading to the nearly complete absence of the fluorescence signal at 520 nm.

Using the Stern–Volmer plot, fluorescence titration was conducted to investigate the quenching mechanisms between the self-assembled peptide amyloid material and uranyl ions (Fig. 10B). According to the experimental results, the Stern–Volmer plot exhibited linearity at lower peptide concentrations. At lower concentrations of the peptide, up to 1 : 1 uranyl to peptide ratio, the Stern–Volmer plot showed a linear trend with respect to an increasing concentration of peptide as a quencher, indicating static quenching due to the fluorophore-quencher or uranyl-peptide ground-state charge-transfer complex formation-based quenching phenomenon. At higher concentrations of the peptide, the deviation from linearity in the Stern–Volmer plot indicates the involvement of a dynamic quenching process.^50^

The Stern–Volmer quenching constant of *K*_SV_ = 1.47 × 10^4^ M^−1^ obtained from the linear region of the (*F*_0_/*F*) *vs.* [*Q*] plot, where *F*_0_, *F*, and [*Q*] are the fluorescence intensity of uranyl nitrate in the absence of quencher, the fluorescence intensity in the presence of different concentrations of peptide amyloid and concentration of peptide amyloid (μM), respectively. The binding ratio of uranium to peptide and its association constant (*K*_A_) were explored employing Benesi–Hildebrand plots for 1 : 1 and 1 : 2 peptide-to-ligand binding ratios (Fig. 10C and S9B†)^51^ and it was found that 1 : 1 metal-to-peptide binding was more prominent according to the best linear fit profile. According to the [(*F*_0_ − *F*_m_)/(*F*_0_ − *F*)] *vs.* 1/[*Q*] plot, where *F*_m_ is the fluorescence intensity in the presence of excess peptide quencher, the uranium ion binds to the peptide with an association constant of *K*_A_ = 1.03 × 10^4^ M^−1^.

#### 3.3.3 Uranium and peptide binding – XPS spectroscopy studies

**Fig. 11:**
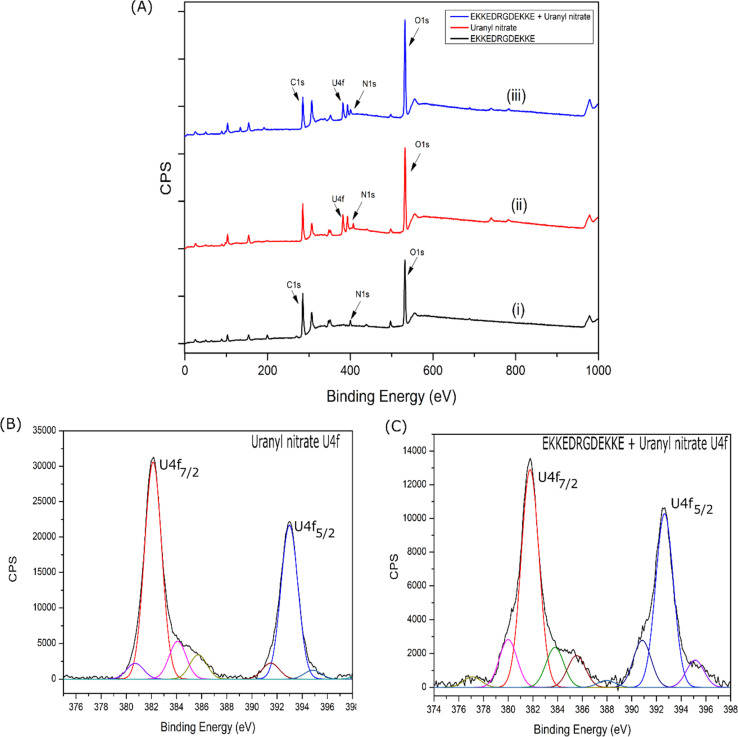
(A) XPS survey spectra for (i) EKKEDRGDEKKE peptide alone, (ii) uranyl nitrate alone, and (iii) EKKEDRGDEKKE–uranyl complex. Narrow band XPS U 4f spectra of uranyl nitrate alone (B) and incubated with EKKEDRGDEKKE peptide (C).

The XPS study was performed to gain insight into the possible mechanism of uranium binding to the EKKEDRGDEKKE amyloids. The uranyl–peptide amyloid complex for the XPS study was prepared by incubating 300 μL of 60 μM (pH 11.7) peptide amyloid solution with 10 μL of 25 μM uranyl nitrate solution overnight and 5 μL of the peptide–uranium complex was drop-casted on a glass slide. The XPS survey scan, as seen in Fig. 11A, illustrates the presence of C, N, and O atoms for the EKKEDRGDEKKE self-assembled peptide and the co-existence of C, N, O, and U for the EKKEDRGDEKKE–uranyl complex. The X-ray photoelectron spectroscopy (XPS) measurements of free uranyl nitrate (U^6+^) indicated the presence of U 4f_7/2_ and U 4f_5/2_ peaks at 382.12 eV and 393.01 eV, respectively^81^ (Fig. 11B). Uranyl nitrate incubated with the EKKEDRGDEKKE peptide amyloid at pH 11.7 exhibited a slight shift in the XPS peaks corresponding to U 4f_7/2_ and U 4f_5/2_ towards lower binding energies, that is, at 381.67 eV and 392.63 eV, respectively (Fig. 11C). These peak shifts indicate the potential binding of the peptide to the uranyl metal center due to the apparent formation of the uranyl–peptide complex. The lower energy small intensity peak observed at 380.78 eV for free uranyl nitrate and at 380.01 eV for uranyl–peptide complexes can be attributed to the U^5+^ state (Fig. 11B and C, respectively) (Table 2). The ratio between U^6+^/U^5+^ in the free uranyl nitrate and uranyl–peptide complex is 13.79 and 4.42, respectively. The X-ray-based reduction process may initiate the formation of U^5+^ from U^6+^. A narrow-band XPS U 4f scan was performed after irradiating the uranyl–peptide complex with X-rays to confirm the reduction of the uranium center induced by X-ray exposure over 10 min. It was found that the intensities of the U^5+^ XPS peak increased in comparison to that of U^6+^ (Fig. S10†).

**Table 2:** XPS U 4f deconvoluted peak binding energy (eV) for uranyl nitrate and the EKKEDRGDEKKE peptide (pH 11.7)–uranyl nitrate complex

Uranyl nitrate		Uranyl nitrate + EKKEDRGDEKKE (pH 11.7)	
U 4f_(7/2)_	U 4f_(5/2)_	U 4f_(7/2)_	U 4f_(5/2)_
380.78 eV (U^5+^)	391.57 eV (U^5+^)	380.01 eV (U^5+^)	390.86 eV (U^5+^)
382.12 eV (main U^6+^)	393.01 eV (main U^6+^)	381.67 eV (main U^6+^)	392.63 eV (main U^6+^)
384.10 eV (satellite)	394.88 eV (satellite)	383.74 eV (satellite)	395.14 eV (satellite)
385.77 eV (satellite)		385.44 eV (satellite)	

The major O 1s XPS peak maximum of uranyl nitrate, EKKEDRGDEKKE (pH = 11.7), and uranyl–peptide complex was observed at 532.2 eV, 532.0 eV, and 531.9 eV (Fig. S11A–C†), respectively. The O 1s XPS features of the uranyl–peptide complex were deconvoluted and assigned to specific chemical functional groups, as shown in Table S7.† The peaks observed at approximately 530.70 eV, 531.86 eV, 531.96 eV, and 533.72 eV are attributed to the deprotonated carboxylate side chain groups of the peptide (associated with E and D amino acids), oxygen atoms of the uranyl group, amide groups of the peptide, and carboxylate groups of the peptide side chains bound to the uranyl moiety, respectively^82^ (Table S7†).

The N 1s XPS peak of the EKKEDRGDEKKE amyloid peptide at pH 11.7 was observed at 399.7 eV. In contrast, the peak maximum for the uranyl–EKKEDRGDEKKE peptide complex was slightly higher at 400.1 eV (Fig. S11D–F†). The N 1s XPS peak of the nitrate group of uranyl nitrate was observed at 407.2 eV (the peaks at around 398.9 eV and 403.5 eV are due to the U 4f satellites), but it was absent in the uranyl–peptide amyloid complex. This absence of an N 1s XPS peak at around 407 eV in the uranyl–peptide complex suggests that the peptide amyloid substituted the nitrate ion, which serves as a coordinating ligand and helps maintain the charge balance.^83^ The N 1s XPS deconvoluted peaks of the EKKEDRGDEKKE peptide amyloid at pH 11.7 were observed at 399.7 eV and 397.8 eV, which are attributed to the amide-N and amine-N groups, respectively. The N 1s XPS spectrum of the uranyl–EKKEDRGDEKKE peptide complex revealed distinct peaks at 401.9 eV, 400.1 eV, and 398.2 eV (Table S7†). These peaks correspond to the uranyl metal center-bound amine groups of the peptide side chain, the amide N atoms, and the uncoordinated side chain amine groups of the peptide, respectively.

**Scheme 3:**
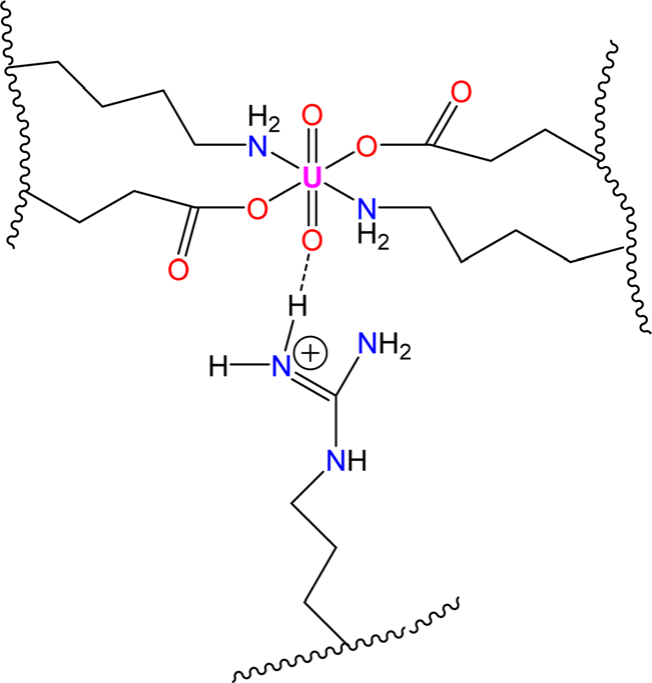
Schematic illustration of coordination environment of uranyl–peptide amyloid complex.

The spectroscopic investigations indicated the formation of a uranyl–EKKEDRGDEKKE peptide amyloid complex possibly adopting a tentative octahedral coordination environment involving the amine groups of the lysine residues and the carboxylate groups of the glutamic acid and/or aspartic acid side chains and additional stability for the complex structure provided by the arginine group through a hydrogen bonding interaction (N–H⋯O=U) between the NH group of arginine and uranyl oxygen atom (Scheme 3).

## 4 Conclusions

The designed EKKEDRGDEKKE dodecapeptide possessed inherent ability to undergo self-assembly and form amyloid nanostructures. It retained the propensity to self-assemble, similar to the exclusively charged polar EKKE peptide. Consequently, the morphology of the self-assembled peptide EKKEDRGDEKKE was influenced by pH and the charge it acquires, which is comparable to the EKKE peptide. These findings highlight and reinforce the significance of the EKKE motif in the fundamental process of self-assembly. Incorporating the DRGD motif also maintained the characteristic morphology of the EKKE peptide under three different pH conditions. The formation of ribbon-like structures at neutral pH and branched forms at basic pH was consistent. However, only aggregates appeared at acidic pH, underscoring the significance of pH and charge in the self-assembly of peptide amyloid materials. The EKKEDRGDEKKE peptide formed thread-like amyloid morphologies at pH 7.4 and fractal-like amyloid nanostructures at 11.7, which is probably due to the difference in the contribution of the β-sheet, α-helix, and β turn regions to the overall formation of nanostructures. The designed self-assembled EKKEDRGDEKKE peptide also showed the ability to bind to lead and uranium ions effectively under basic (pH 11.7) conditions, as demonstrated by UV absorption, atomic absorption, and XPS spectroscopy, thus establishing the role played by the introduced DRGD scaffold in chelating the toxic metal ions. The fractal morphology of this apo-peptide amyloid slightly changed to a more open fractal morphological structure upon uranium binding and slightly leaf-like fractal structure in the lead–peptide amyloid complex. Hence, the novel-designed EKKEDRGDEKKE peptide not only retained the self-assembling potential of the EKKE motif but could also bind to the toxic lead metal ion, and additionally uranium metal ions. The pH conditions influenced the binding of the heavy toxic metal ions to the self-assembled peptide amyloid material. Notably, an alkaline pH of 11.7 resulted in enhanced binding capabilities compared to the acidic pH level of 3.6 and a neutral pH of 7.4. The binding of the metal ion was also concentration dependent, indicating the role played by the residues in the self-assembled peptide amyloid material in the coordination of the toxic metal ions. Incorporating the DRGD motif facilitated the presence of specific residues (arginine, glycine, and aspartic acid) and a suitable surface for the interaction between the peptide-amyloid material and the metal ion. Also, it was clear that the coordination of lead and uranium metal ions was facilitated by the O and N atoms, as evidenced by the change in their binding energy (eV) values in the X-ray photoelectron spectra. The role of the DRGD motif can further be extended to other applications, such as anti-microbial studies, tissue engineering, and other biomedical applications, rendering this peptide a unique multi-functionality.

## Abbreviations

E: Glutamic acid
K: Lysine
D: Aspartic acid
R: Arginine
G: Glycine
ThT: Thioflavin T
CD: Circular dichroism
HFIP: 1,1,1,3,3,3-Hexafluoro-2-propanol
FE-SEM: Field Emission Scanning Electron Microscopy
AFM: Atomic Force Microscopy
UV: Ultra-Violet
XPS: X-ray Photoelectron Spectroscopy
CPS: Counts per second
eV: Electron volts

## Author contributions

Aishwarya Natarajan: investigation, methodology, formal analysis, data curation, validation, writing – original draft, writing – review and editing. Ramakrishna Vadrevu: conceptualization, funding acquisition, methodology, resources, supervision. Krishnan Rangan: conceptualization, funding acquisition, data curation, formal analysis, investigation, methodology, project administration, resources, supervision, writing – original draft, writing – review and editing.

## Conflicts of interest

The authors affirm that they do not possess any identifiable personal or competing conflicts of interest that might have appeared to influence the research presented in this article.

## Supplementary Material

RA-014-D3RA08261J-s001
