# Supplementary material for: DRGD-linked charged EKKE dimeric dodecapeptide: pH-based amyloid nanostructures and their application in lead and uranium binding
Source: RSC Adv. 2024 Mar 19;14(13):9200–17. doi: 10.1039/d3ra08261j (PMC10949120; doi:10.1039/d3ra08261j)
Supplement: RA-014-D3RA08261J-s001 [file RA-014-D3RA08261J-s001.pdf]

Electronic Supplementary Material (ESI) for RSC Advances.
This journal is © The Royal Society of Chemistry 2024

DRGD linked charged EKKE dimeric dodecapeptide: pH-based amyloid nanostructures and their application in lead and uranium binding

Aishwarya Natarajan[1,2], Late Ramakrishna Vadrevu[2], Krishnan Rangan[1*]

[1]Department of Chemistry, Birla Institute of Technology and Science Pilani, Hyderabad Campus, Jawahar Nagar, Hyderabad 500 078 Telangana, India

[2]Department of Biological Sciences, Birla Institute of Technology and Science Pilani, Hyderabad Campus, Jawahar Nagar, Hyderabad 500 078 Telangana, India

Email: rkrishnan@hyderabad.bits-pilani.ac.in

*Corresponding Author

Supplementary Data

HPLC REPORT

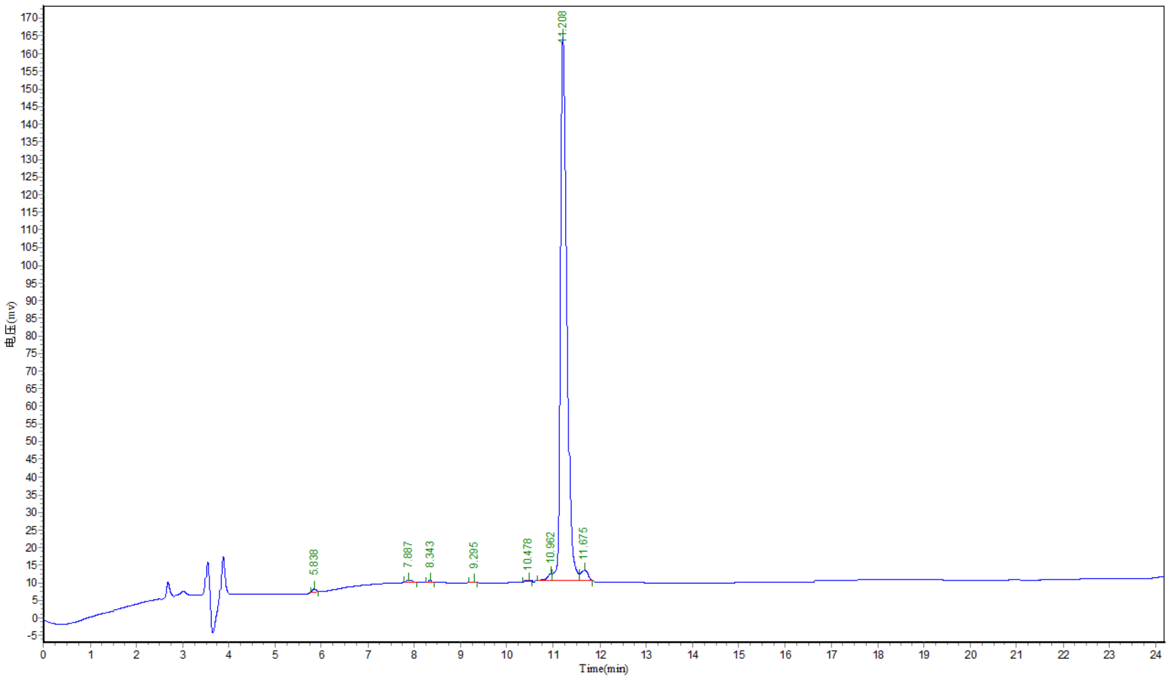

Sample: Ac-EE-12 Analyzed date: 2019-3-16
Lot. No.: P190307-MX712437
Column: Kromasil-C18, 4.6*250mm, 5μm
Solvent A A: 0.1% Trifluoroacetic Acid in 100% Acetonitrile
Solvent B B: 0.1% Trifluoroacetic Acid in 100% Water

	A	B
0.0min	6%	94%
25.0min	31%	69%
25.1min	100%	0%
30.0min		Stop

Volume: 10μl
Wavelength: 220nm
Flow rate: 1.0ml/min

Figure S1: HPLC profile of Ac-EKKEDRGDEKKE-$CONH_2$

MASS SPECTROMETRY REPORT

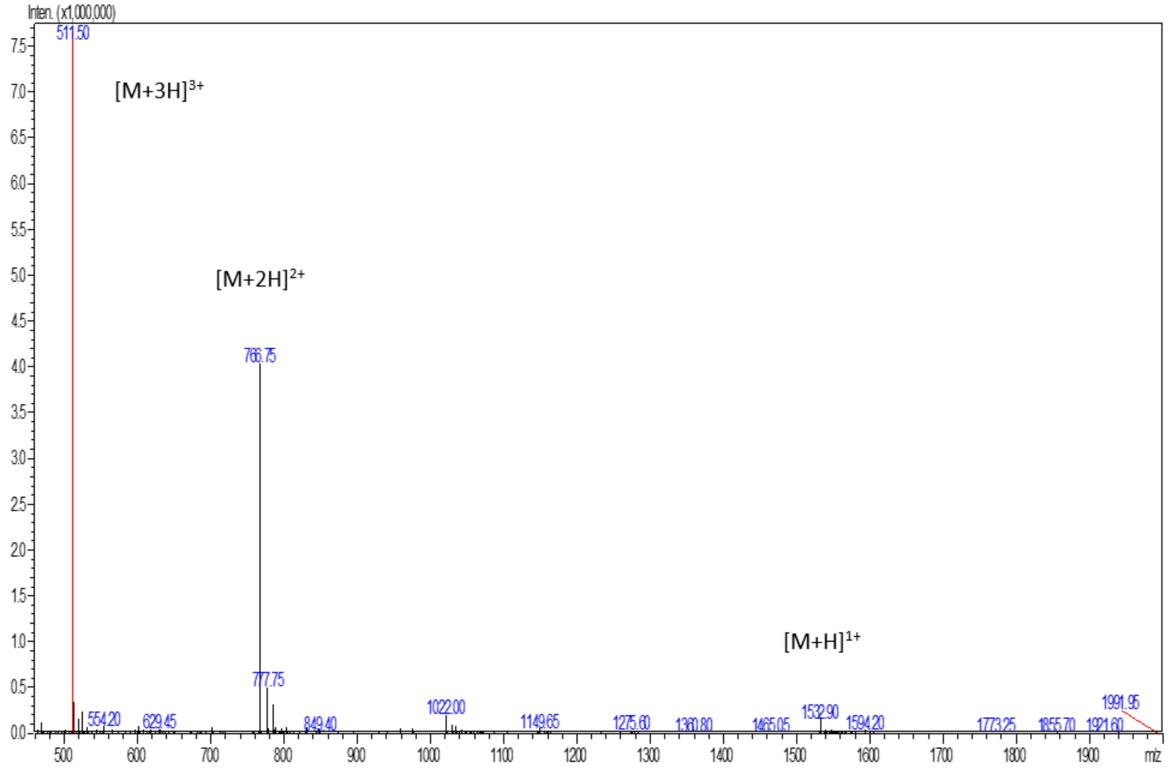

Sample Description		Instrument	SHIMADZU LCMS-2020		
Analyzed date:	2019/3/15	Probe:	ESI	Probe Bias:	＋4.5kv
Analyst:	Shen	Nebulizer Gas Flow:	1.5L/min	Detector:	1.2kv
Sample:	Ac-EE-12	CDL:	-20.0v	T. Flow:	0.2ml/min
M.W.:	1531.66	CDL Temp.:	250 °C	B. Conc.:	50%H2O/50%ACN
Lot. No.:	P190307-MX712437	Block Temp.:	400 °C		

Figure S2: Mass spectrometry report of Ac-EKKEDRGDEKKE-$CONH_2$

Table S1: DLS size profile for Ac-EKKEDRGDEKKE-CONH$_2$ peptide at pH 3.6, 7.4 and 11.7 at 260μM concentration.

EKKEDRGDEKKE peptide	1h size profiles (Z-average)	3 days size profiles (Z-average)
pH 3.6	181.8 d.nm, 242.5 d.nm, 226.9 d.nm (PdI 0.316)	429.1 d.nm, 432 d.nm, 596.9 d.nm (PdI 0.334)
pH 7.4	113.1 d.nm, 196.9 d.nm, 370 d.nm (PdI 0.471)	412.5 d.nm, 465.8 d.nm, 570 d.nm (PdI 0.515)
pH 11.7	285.73 d.nm, 356.3 d.nm, 372.1 d.nm (PdI 0.389)	413.1 d.nm, 459.8 d.nm, 552.4 d.nm (PdI 0.496)

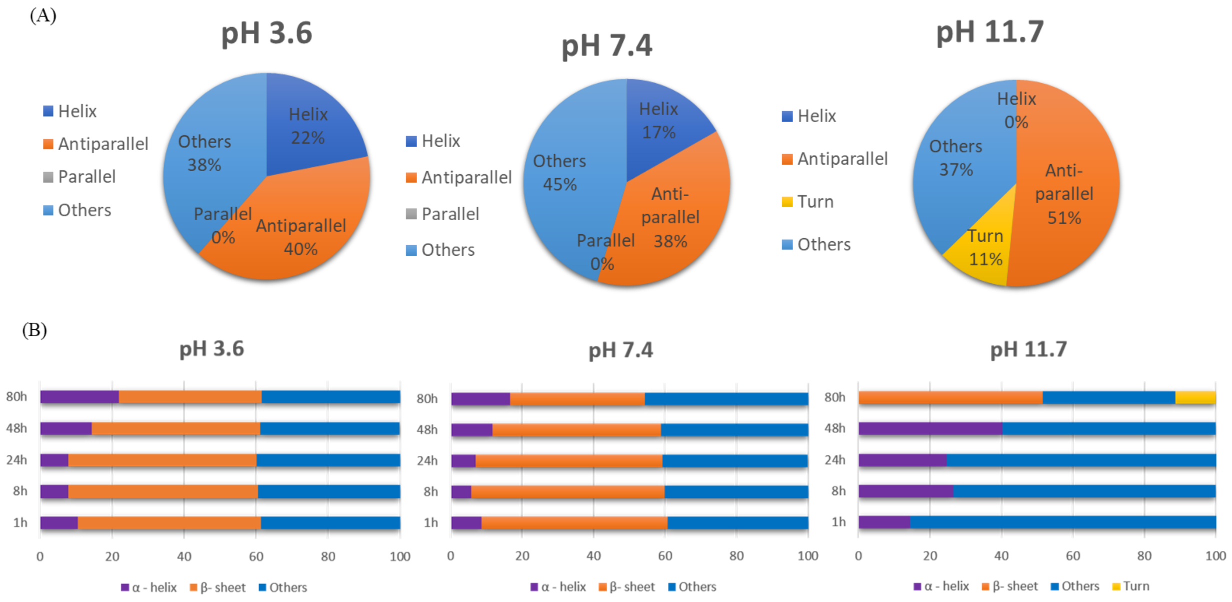

Figure S3: [A] Amount of secondary structure elements present at 80 h post dissolution of EKKEDRGDEKKE peptide at pH 3.6, 7.4 and 11.7 and [B] time dependent representation (in %) of the different secondary structure elements for the peptide EKKEDRGDEKKE from the point of incubation at 1h, 8h, 24h, 48h and 80h.

Table S2 (% of secondary structure elements in EKKEDRGDEKKE peptide): *Percentage of secondary structure elements present in EKKEDRGDEKKE peptide at the three pH conditions, pH 3.6, pH 7.4 and pH 11.7 across time intervals, 1h, 8h, 24h, 48h and 80h obtained from BestSel analysis of circular dichroism information XPS of peptide and Pb-complexes at three pHs.*

Time	EKKEDRGDEKKE pH 3.6	EKKEDRGDEKKE pH 7.4	EKKEDRGDEKKE pH 11.7
**1 h**	α-helix = 10.5 %<br>β-sheets = 50.8 %<br>Others = 38.7 %	α-helix = 8.7 %<br>β-sheets = 52 %<br>Others = 39.3 %	α-helix = 14.5 %<br>β-sheets = 0 %<br>Others = 88.5 %
**8 h**	α-helix = 7.8 %<br>β-sheets = 52.8 %<br>Others = 39.4 %	α-helix = 5.7 %<br>β-sheets = 54.2 %<br>Others = 40.2 %	α-helix = 26.5 %<br>β-sheets = 0 %<br>Others = 73.5 %
**24 h**	α-helix = 7.8 %<br>β-sheets = 52.4 %<br>Others = 39.8 %	α-helix = 6.9 %<br>β-sheets = 52.4 %<br>Others = 40.6 %	α-helix = 24.7 %<br>β-sheets = 0 %<br>Others = 78.3 %
**48 h**	α-helix = 14.4 %<br>β-sheets = 46.7 %<br>Others = 38.8 %	α-helix = 11.8 %<br>β-sheets = 47.1 %<br>Others = 41.1 %	α-helix = 40.3 %<br>β-sheets = 0 %<br>Others = 59.7 %
**80 h**	α-helix = 21.9 %<br>β-sheets = 39.6 %<br>Others = 38.5 %	α-helix = 16.7 %<br>β-sheets = 37.7 %<br>Others = 45.6 %	α-helix = 0 %<br>β-sheets = 51.5 %<br>Others = 37.2 %<br>β-turn = 11.2 %

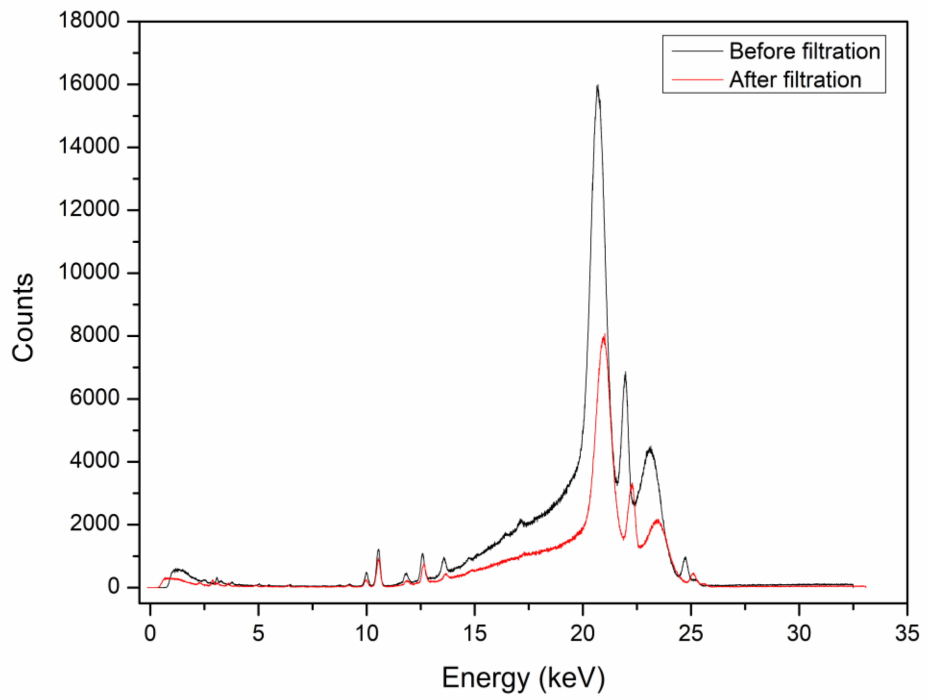

Figure S4: XRF of mixed metal ions (100 ppm of each vanadyl sulfate, cadmium chloride, mercury chloride, lead acetate, uranyl nitrate) before and after treated with EKKEDRGDEKKE peptide amyloids at pH 11.7.

Table S3: *Relative concentrations of cadmium (Cd^{2+}), mercury (Hg^{2+}), lead (Pb^{2+}), uranyl ($U(O)_2^{2+}$) and vanadyl ($V(O)^{2+}$) ions in solution before and after incubation with the EKKEDRGDEKKE peptide amyloids at pH 11.7.*

Metal	***Before filtration***	***After filtration***
Vanadium	*20.258%*	*25.949%*
Cadmium	*13.136%*	*16.274%*
Mercury	*17.701%*	*30.845%*
Lead	*23.032%*	*15.566%*
Uranium	*25.832%*	*11.367%*
Total	*100%*	*100%*

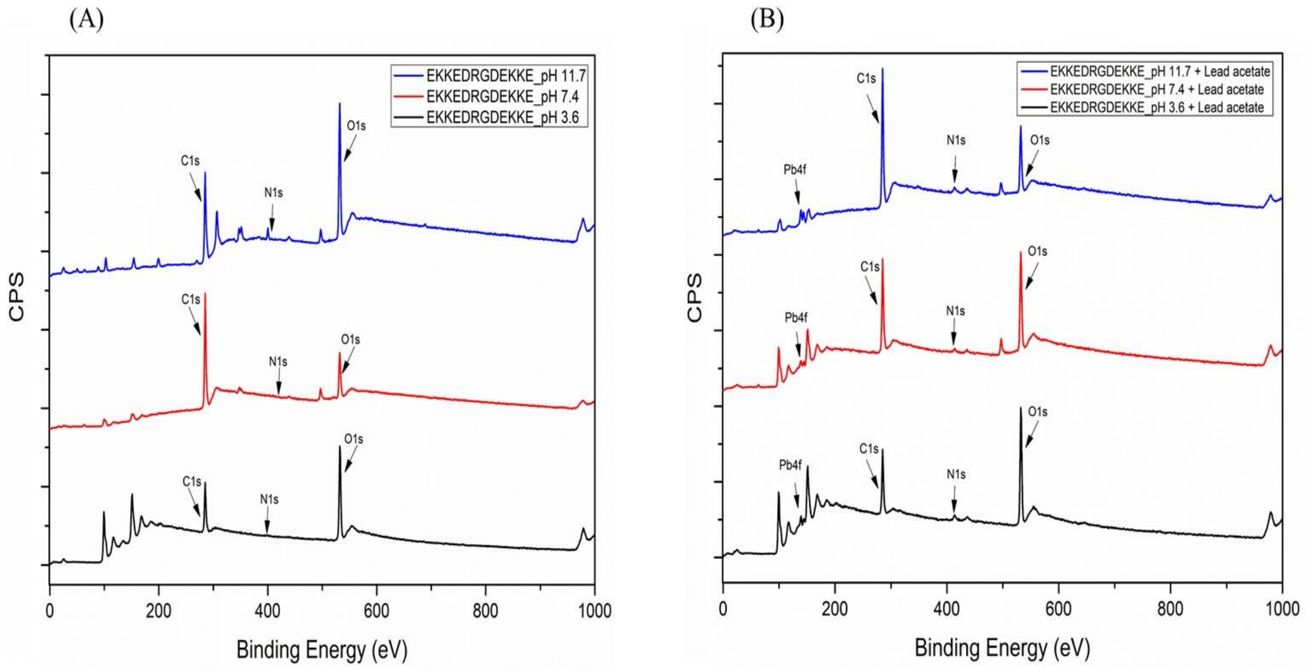

Figure S5: [A] XPS survey spectra of EKKEDRGDEKKE self-assembled amyloid peptide at the three different pH conditions, pH 3.6, 7.4 and 11.7. [B] XPS survey spectra of EKKEDRGDEKKE self-assembled amyloid peptide incubated with lead acetate metal solution at three different pH conditions, pH 3.6, 7.4 and 11.7.

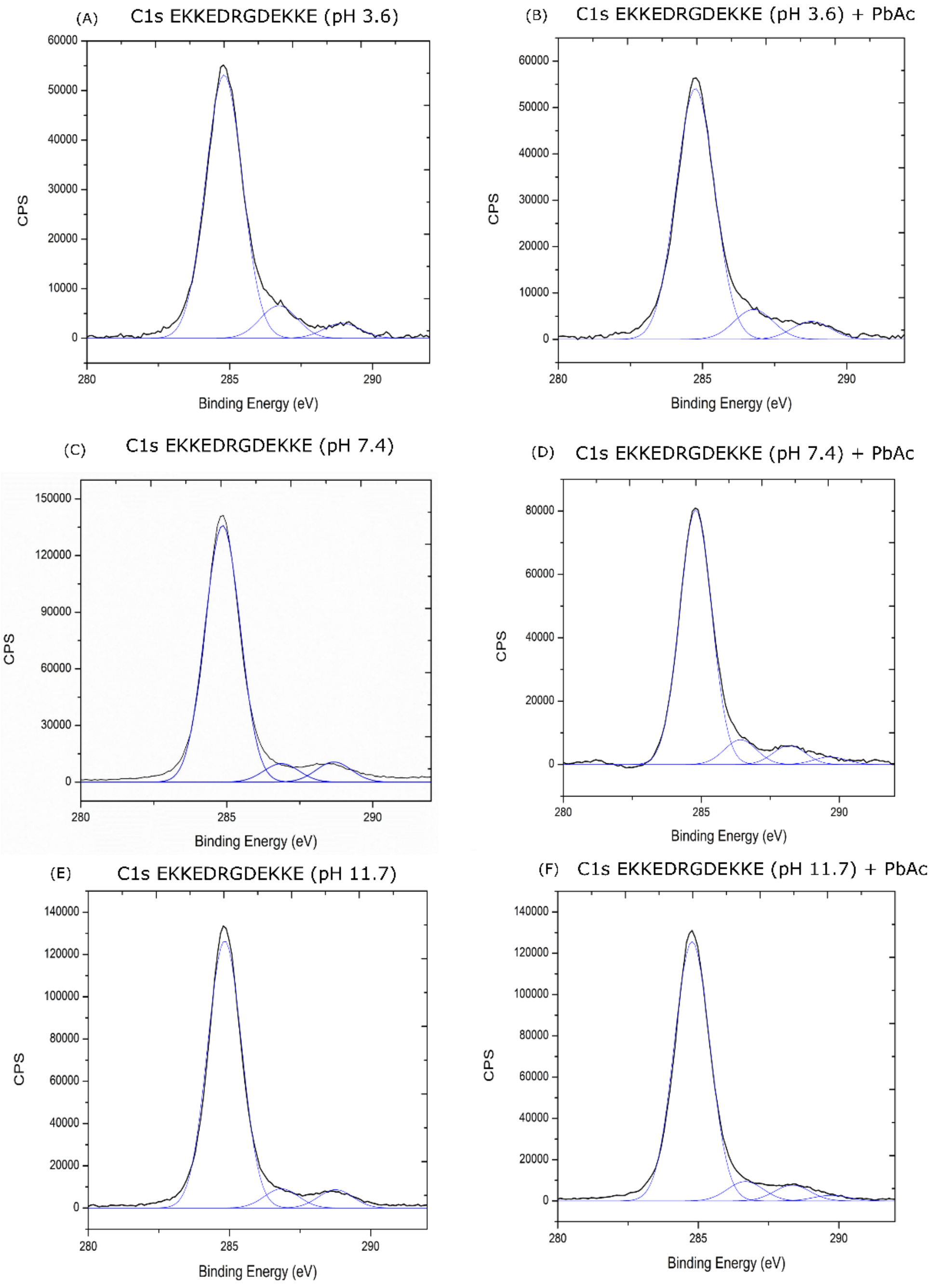

Figure S6: (C1s XPS of peptide and Pb-complexes at three pHs): C1s spectra of (a) EKKEDRGDEKKE self-assembled peptide amyloid at pH 3.6 (b) EKKEDRGDEKKE self-assembled peptide amyloid at pH 3.6 and 5ppm lead acetate (c) EKKEDRGDEKKE self-assembled peptide amyloid at pH 7.4 (d) EKKEDRGDEKKE self-assembled peptide at pH 7.4 (d) EKKEDRGDEKKE self-assembled peptide amyloid at pH 7.4 and lead acetate (e) EKKEDRGDEKKE self-assembled amyloid at pH 11.7 (f) EKKEDRGDEKKE self-assembled peptide amyloid at pH 11.7 and 5ppm lead acetate.

Table S4 (C1s XPS of peptide and Pb-complexes at three pHs)*: C1s binding energy (eV) values for self-assembled EKKEDRGDEKKE peptide and EKKEDRGDEKKE peptide and lead metal complex.*

**C1s values for EKKEDRGDEKKE peptide**				**C1s values for EKKEDRGDEKKE peptide and lead metal**			
**Functional Group C(1s)**	**pH 3.6**	**pH 7.4**	**pH 11.7**	**Functional Group C(1s)**	**pH 3.6 + Lead**	**pH 7.4 + Lead**	**pH 11.7 + Lead**
**Hydrocarbon**	284.8 eV	284.8 eV	284.8 eV	**Hydrocarbon**	284.7 eV	284.8 eV	284.8 eV
**amine(C-N) and alpha carbon (C-N-CO) carboxylate (O=C-O$^-$)**	286.7 eV	286.8 eV	286.8 eV	**amine(C-N) and alpha carbon (C-N-CO)**	286.7 eV	286.4 eV	286.6 eV
**amide ((C=O)-N), and carboxylic acid (O=C-OH)**	288.9 eV	288.7 eV	288.7 eV	**amide ((C=O)-N), carboxylate (O=C-O$^-$) and carboxylic acid (O=C-OH)**	288.8 eV	288.1 eV	288.3 eV
				**Lead bound carboxylate (O=C-O-Pb)**		289.6 eV	289.8 eV

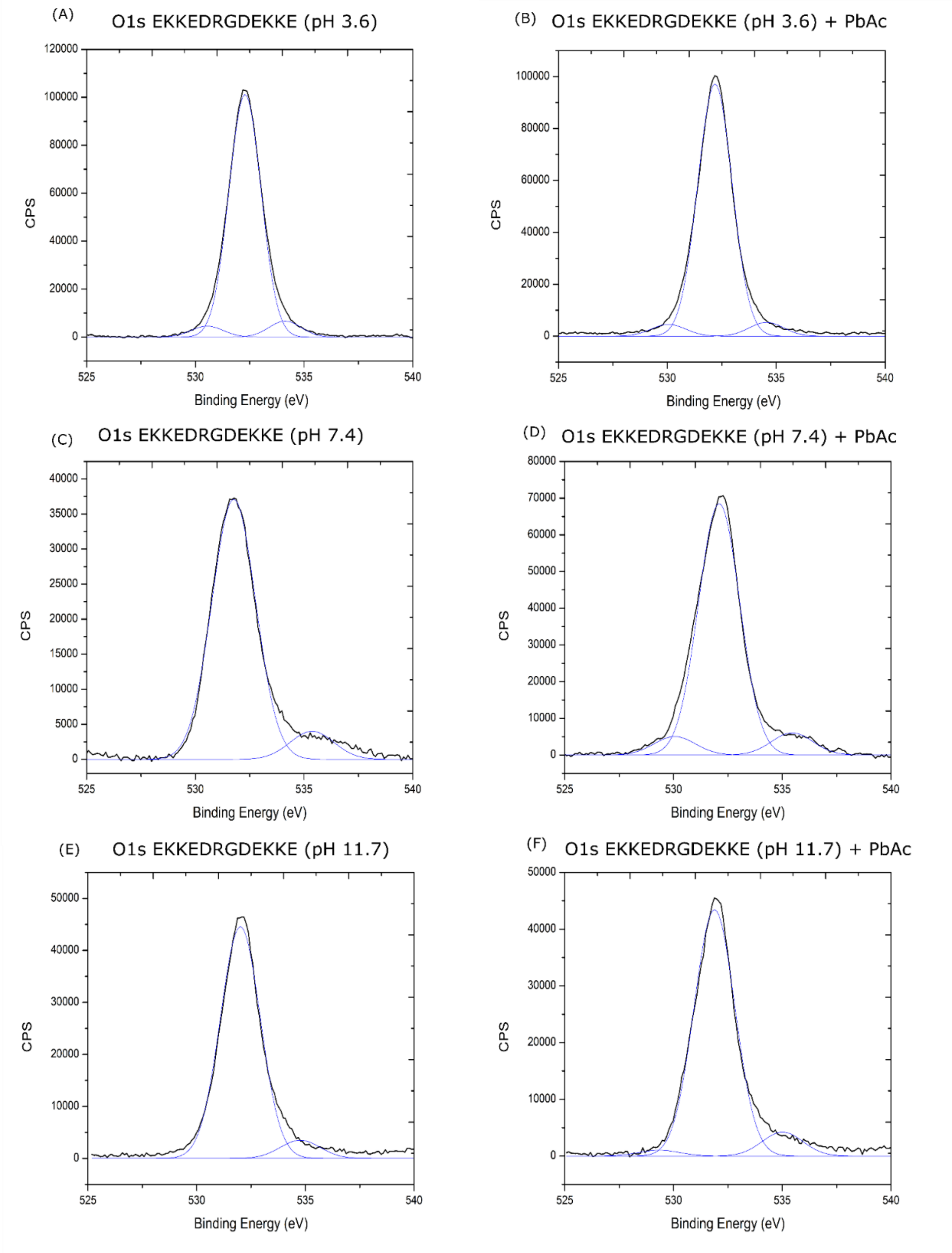

Figure S7: (O1s XPS of peptide and Pb-complexes at three pHs): O1s spectra of (a) EKKEDRGDEKKE self-assembled peptide amyloid at pH 3.6 (b) EKKEDRGDEKKE self-assembled peptide amyloid at pH 3.6 and 5ppm lead acetate (c) EKKEDRGDEKKE self-assembled peptide amyloid at pH 7.4 (d) EKKEDRGDEKKE self-assembled peptide at pH 7.4 and 5ppm lead acetate (e) EKKEDRGDEKKE self-assembled peptide amyloid at pH 11.7 (f) EKKEDRGDEKKE self-assembled peptide amyloid at pH 11.7 and 5ppm lead acetate.

Table S5 (O1s XPS of peptide and Pb-complexes at three pHs). *O1s binding energy (eV) values for self-assembled EKKEDRGDEKKE peptide and EKKEDRGDEKKE peptide and lead metal complex.*

**O1s values for EKKEDRGDEKKE peptide**				**O1s values for EKKEDRGDEKKE peptide and lead metal**			
**Functional Group O(1s)**	**pH 3.6**	**pH 7.4**	**pH 11.7**	**Functional Group O(1s)**	**pH 3.6 + Lead**	**pH 7.4 + Lead**	**pH 11.7 + Lead**
**Carboxylate ($O{=}C{-}O^-$)**				**Carboxylate ($O{=}C{-}O^-$)**			529.3 eV
**Carboxylic acid (O=C-OH)**	530.5 eV			**Carboxylic acid (O=C-OH)**	530.0 eV	530.6 eV	
**Amide (O=C-NH-)**	532.2 eV	531.7 eV	532.0 eV	**Amide (O=C-NH-)**	532.2 eV	532.1 eV	531.8 eV
**Carboxylic acid (HO-C=O)**	534.1 eV			**Carboxylic acid (HO-C=O)**	534.5 eV		
**Carboxylate-Na^+**		535.3 eV	534.4 eV	**Carboxylate-Na^+**			
				**Carboxylate-Pb^{2+}**		535.4 eV	535.0 eV

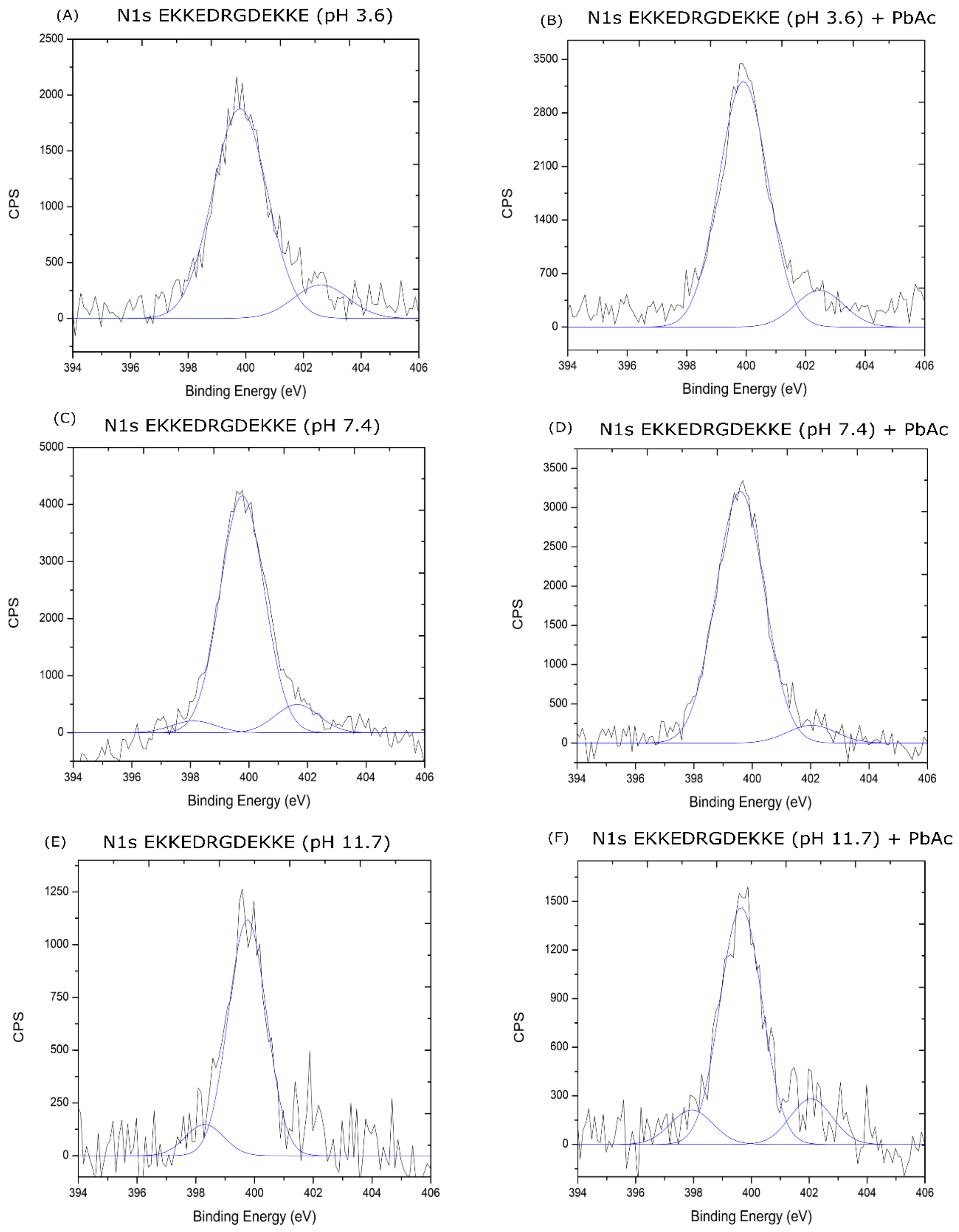

Figure S8: N1s spectra of (A) EKKEDRGDEKKE self-assembled peptide amyloid at pH 3.6 (B) EKKEDRGDEKKE self-assembled peptide amyloid at pH 3.6 and 5ppm lead acetate (C) EKKEDRGDEKKE self-assembled peptide amyloid at pH 7.4 (D) EKKEDRGDEKKE self-assembled peptide at pH 7.4 and 5ppm lead acetate (E) EKKEDRGDEKKE self-assembled peptide amyloid at pH 11.7 (F) EKKEDRGDEKKE self-assembled peptide amyloid at pH 11.7 and 5ppm lead acetate.

Table S6: (N1s XPS of peptide and Pb-complexes at three pHs). *N1s binding energy (eV) values for self-assembled EKKEDRGDEKKE peptide and EKKEDRGDEKKE peptide and lead metal complex.*

**N1s values for EKKEDRGDEKKE peptide**				**N1s values for EKKEDRGDEKKE peptide and lead metal**			
**Functional Group N(1s)**	**pH 3.6**	**pH 7.4**	**pH 11.7**	**Functional Group N(1s)**	**pH 3.6**	**pH 7.4**	**pH 11.7**
**Amine (C-NH$_2$)**		398.1 eV	397.8 eV	**Amine (C-NH$_2$)**			397.6
**Amide (O=C-NH-C)**	399.8 eV	399.7 eV	399.7 eV	**Amide (O=C-NH-C)**	399.8	399.6	399.6
**Amine+H$^+$ ion (R-NH$_3^+$)**	402.5 eV	401.6 eV		**Amine+H$^+$ ion (R-NH$_3^+$)**	402.5		
				**Amine + Pb^{2+}**		402.0	402.0

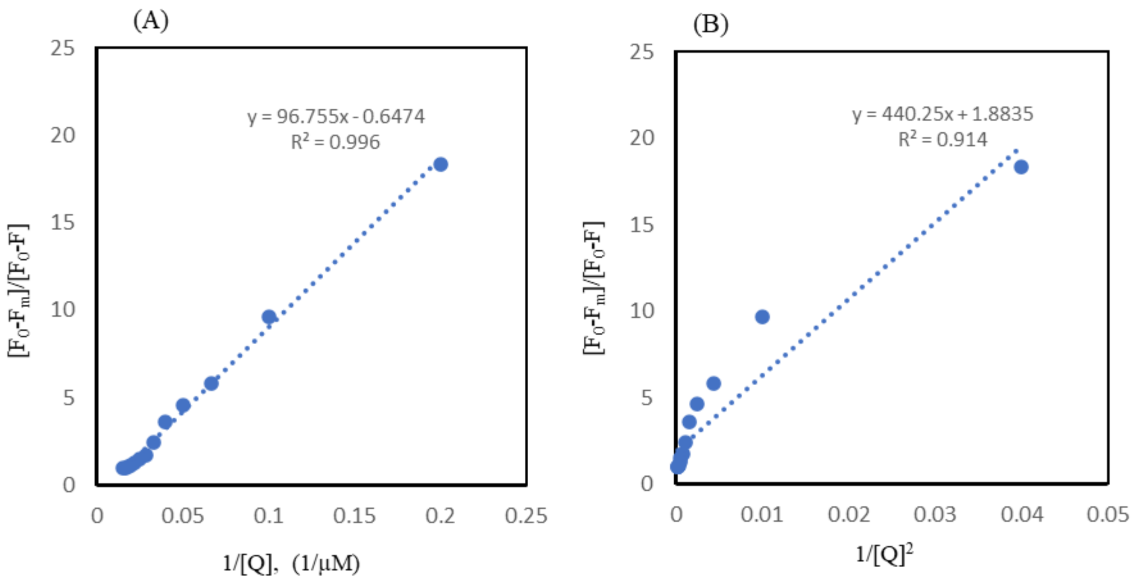

Figure S9: Benesi-Hildebrand plot of [(F_0-F_m)/(F_0-F)] (F_0 is the fluorescence intensity without the peptide and F_{min} is the minimum fluorescence intensity upon quenching), versus (a) [1/Q] (Q= peptide concentration in μM) showcasing 1:1 metal and peptide binding. and (b) [$1/Q^2$] (Q= peptide concentration) representing 1:2 metal and peptide binding.

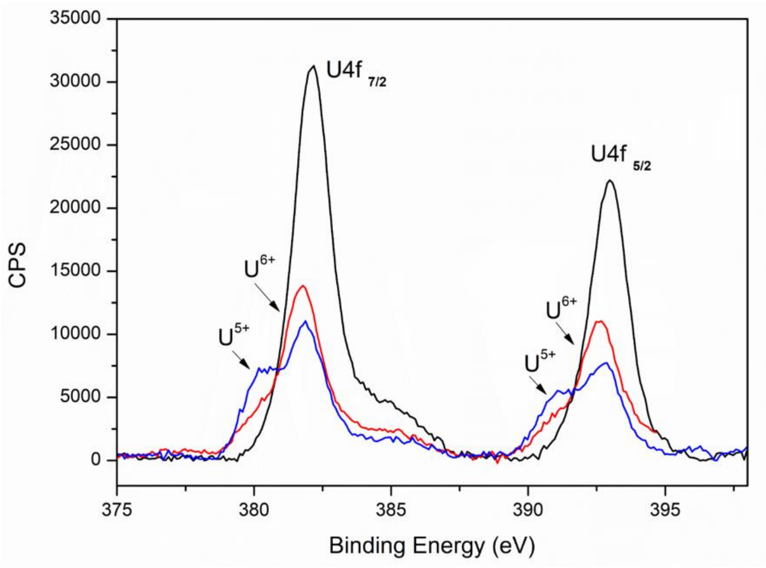

Figure S10: U4f XPS spectra of uranyl nitrate (black) at U^{6+} oxidation state, uranyl nitrate - EKKEDRGDEKKE complex (red) and uranyl nitrate EKKEDRGDEKKE complex after x-ray exposure for 10 minutes (blue) at U^{5+} oxidation state.

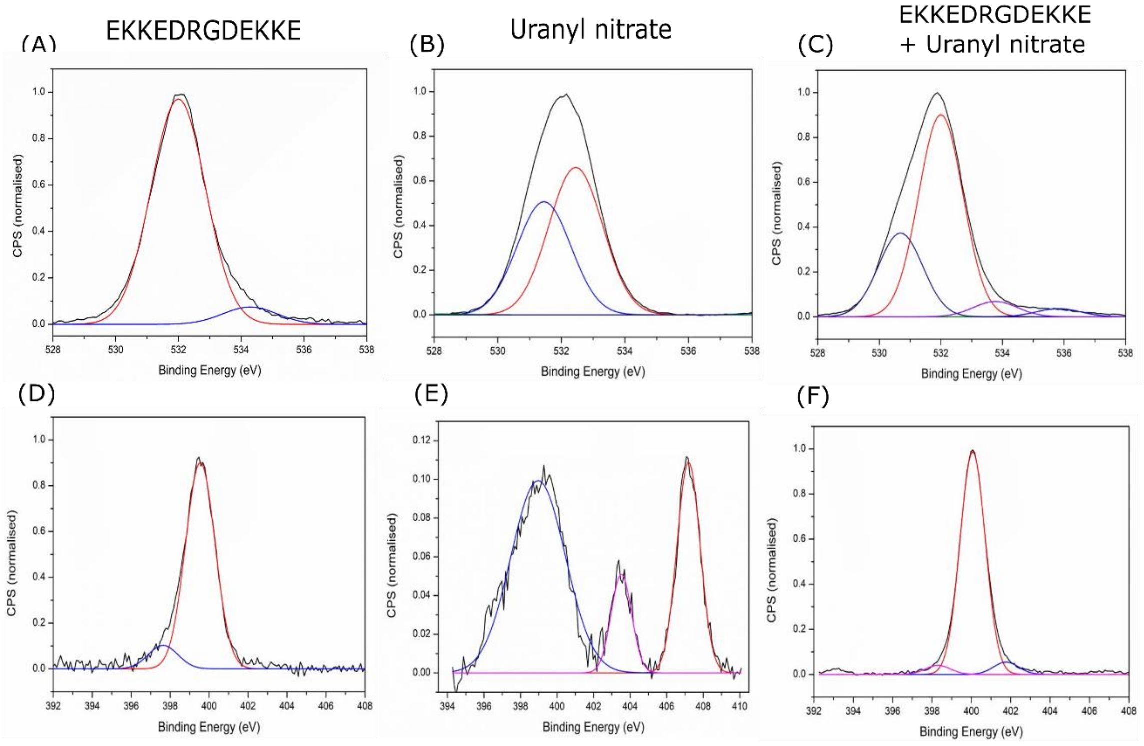

Figure S11: Deconvoluted XPS spectra of O and N atoms of uranyl nitrate, EKKEDRGDEKKE peptide and EKKEDRGDEKKE peptide incubated with uranyl nitrate to represent the various contributions of the various bonds based on the binding energy (eV) values.

Table S7: (O1s N1s XPS of peptide and U-complex). *O1s and N1s binding energy (eV) values for uranyl nitrate, self-assembled EKKEDRGDEKKE peptide, and EKKEDRGDEKKE peptide - uranyl nitrate complex.*

**Sample**	**O1s**	**N1s**
Uranyl nitrate	531.56 eV (U=O);<br>532.56 eV (NO_3^-);<br>532.21 (major)	407.2 eV (NO_3^-)
EKKEDRGDEKKE	532.01 eV (amide);<br>534.42 eV (carboxylate-Na^+)<br>532.04 eV (major)	399.7 eV (amide);<br>397.8 eV (amine)
Uranyl nitrate+<br>EKKEDRGDEKKE	530.70 eV (carboxylate);<br>531.86 eV (Uranyl);<br>531.96 eV (amide);<br>533.72 eV (carboxylate-uranyl)<br>531.95 (major)	401.9 eV (uranyl bound amine);<br>400.1 eV (amide N);<br>398.2 eV (unbound amine)
